# Functional Annotation of the Transcriptome of the Pig, *Sus scrofa*, Based Upon Network Analysis of an RNAseq Transcriptional Atlas

**DOI:** 10.3389/fgene.2019.01355

**Published:** 2020-02-14

**Authors:** Kim M. Summers, Stephen J. Bush, Chunlei Wu, Andrew I. Su, Charity Muriuki, Emily L. Clark, Heather A. Finlayson, Lel Eory, Lindsey A. Waddell, Richard Talbot, Alan L. Archibald, David A. Hume

**Affiliations:** ^1^ Mater Research Institute-University of Queensland, Translational Research Institute, Woolloongabba, QLD, Australia; ^2^ Nuffield Department of Clinical Medicine, University of Oxford, Oxford, United Kingdom; ^3^ Department of Integrative Structural and Computational Biology, The Scripps Research Institute, La Jolla, CA, United States; ^4^ The Roslin Institute, University of Edinburgh, Midlothian, United Kingdom

**Keywords:** pig, Functional Annotation of Animals Genomes, expression atlas, transcriptomics, network analysis

## Abstract

The domestic pig (*Sus scrofa*) is both an economically important livestock species and a model for biomedical research. Two highly contiguous pig reference genomes have recently been released. To support functional annotation of the pig genomes and comparative analysis with large human transcriptomic data sets, we aimed to create a pig gene expression atlas. To achieve this objective, we extended a previous approach developed for the chicken. We downloaded RNAseq data sets from public repositories, down-sampled to a common depth, and quantified expression against a reference transcriptome using the mRNA quantitation tool, Kallisto. We then used the network analysis tool Graphia to identify clusters of transcripts that were coexpressed across the merged data set. Consistent with the principle of guilt-by-association, we identified coexpression clusters that were highly tissue or cell-type restricted and contained transcription factors that have previously been implicated in lineage determination. Other clusters were enriched for transcripts associated with biological processes, such as the cell cycle and oxidative phosphorylation. The same approach was used to identify coexpression clusters within RNAseq data from multiple individual liver and brain samples, highlighting cell type, process, and region-specific gene expression. Evidence of conserved expression can add confidence to assignment of orthology between pig and human genes. Many transcripts currently identified as novel genes with ENSSSCG or LOC IDs were found to be coexpressed with annotated neighbouring transcripts in the same orientation, indicating they may be products of the same transcriptional unit. The meta-analytic approach to utilising public RNAseq data is extendable to include new data sets and new species and provides a framework to support the Functional Annotation of Animals Genomes (FAANG) initiative.

## Introduction

The domestic pig (*Sus scrofa*) is an important source of meat and other animal products. Because of its human-like size and physiology, the pig (including the mini-pig) is also widely used as an animal model for preclinical studies (Hasenfuss, 1998; Fairbairn et al., 2011; Freeman et al., 2012; Gutierrez et al., 2015) especially since the advent of genome-editing technologies (Whitelaw et al., 2016; Perleberg et al., 2018). In the specific case of the immune system, pigs have provided a useful model for postnatal human development of acquired immunity (Butler et al., 2009); their macrophage-specific gene expression and response to stimulation by toll-like receptor agonists more closely resembles humans than commonly used mouse models (Kapetanovic et al., 2012; Schroder et al., 2012; Kapetanovic et al., 2013). Indeed, better identification of transcription start sites was previously validated by cross-species mapping of the large human FANTOM5 promoter data set (FANTOM Consortium et al., 2014; Robert et al., 2015). The potential for genomic selection and editing for production traits and the utility of the pig as a model organism has recently been expedited by the release of a highly contiguous reference genome (Sscrofa11.1) and parallel sequencing and annotation of a second genome (USMARCv1.0) (Warr et al., 2019). Annotation of expressed sequences and intron-exon boundaries within the genomic sequence was improved through the integration of long read IsoSeq and short-read RNAseq data (Beiki et al., 2019).

Although knowledge of the transcriptomic landscape of the pig is increasing, the functional annotation of the genome remains a work in progress. Many genes in Ensembl are described as “novel pig gene” and annotated solely by a gene number. Around 70-80% of predicted protein-coding genes have been assigned 1:1 orthology with human (Warr et al., 2019) but the NCBI and Ensembl annotations of protein-coding genes do not overlap perfectly. Even where there is 1:1 orthology at the level of protein-coding sequence and conservation of synteny of genomic location with other mammals the expression may not be conserved. Transcriptional regulation has evolved rapidly among mammalian species (Villar et al., 2015; Jubb et al., 2016). Pigs are undoubtedly more human-like than mice (Beiki et al., 2019). Their promoters, identified by cross-mapping of transcription start sites from the human FANTOM5 data set, are more closely related at the DNA sequence level to human (FANTOM Consortium et al., 2014; Robert et al., 2015). But even for those genes for which orthology has been accepted and a gene name assigned, the extent to which expression and function are shared between pigs, humans, and other animals (notably rodent models) remains to be established (Schroder et al., 2012; Jubb et al., 2016). Where expression and function were directly evaluated, for example, in the response of macrophages to lipopolysaccharide, humans and pigs were similar but there were also clear differences (Kapetanovic et al., 2012). What is needed is a resource for pigs comparable to the FANTOM5 (FANTOM Consortium et al., 2014) and GTEx human atlases (GTEx Consortium, 2015). This is a major objective of the international FAANG Consortium (Andersson et al., 2015). By analogy with human data, such a resource could be used to infer the expression profiles of differentiated cell types within tissues even when the cells have not been isolated and profiled directly (Wang et al., 2016).

Although it was once suggested that guilt-by-association is the exception rather than the rule in gene regulatory networks (Gillis and Pavlidis, 2012), on a genome-wide scale across large multitissue data sets the principle is very well established. Genes associated with specific organs, cell types, organelles, and pathways (e.g., the cell cycle, protein synthesis, oxidative phosphorylation/mitochondria) tend to be coexpressed with the transcription factors that regulate them (Hume et al., 2010; Freeman et al., 2012; Mabbott et al., 2013; FANTOM Consortium et al., 2014; Ballouz et al., 2017; Carpanini et al., 2017; Clark et al., 2017; Giotti et al., 2018; Singh et al., 2018). The only significant exception is a subset of genes that appear to have idiosyncratic profiles because they have alternative tissue-specific promoters in some cases encoding different protein isoforms with alternative N-termini. The identification of the coregulated partners of genes with alternative promoters depends upon promoter-based expression profiling (FANTOM Consortium et al., 2014).

In the pig, the principal of coexpression of genes of related function was demonstrated previously using a custom microarray (Freeman et al., 2012), analysing multiple tissues and cells from a single male and female pig of one breed. Among other observations this study identified coexpression of transcripts associated with mitochondria and oxidative phosphorylation and separate regulation of nuclear and mitochondria-encoded transcripts. A more focussed network analysis was used to annotate cell type-specific gene expression in the pig immunome (Dawson et al., 2013). The annotation of the new pig genome assemblies (Sscrofa11.1 and USMARCv1.0) exploited deep RNAseq data from 27 tissues from the reference Duroc breed (BioProject PRJEB19386). The RNAseq data are available in the EBI expression atlas alongside gene expression atlases from other species (Papatheodorou et al., 2018). However, as there are many tissues and cell types missing from that data set, it has limited scope to drive transcriptional network analysis.

There are numerous additional pig RNAseq data sets from diverse tissues, breeds, and conditions in the public domain. We recently developed an approach to harvest and normalize such published data to create an expression atlas for the chicken (Bush et al., 2018). Based upon the use of the high-speed “pseudo-aligner” Kallisto (Bray et al., 2016) to quantify expression this atlas was rapidly created and easily scalable to include new data sets. Here we have used the same atlas-creation pipeline to produce an extended expression atlas for the domestic pig. We used network analysis to identify sets of coexpressed transcripts and present evidence that such analysis can add confidence to orthology and ontology assignments by comparison to human expression data. The pipeline is extensible to provide further refinements to functional annotation in pig and to generate similar resources for other species.

## Methods

### Selecting Samples for an Expression Atlas of the Domestic Pig

We created an expression atlas for the domestic pig by aggregating publicly archived RNAseq libraries, using a pipeline previously described for chickens (Bush et al., 2018). To identify candidate libraries, we downloaded the daily updated SRA BioProject summary file (n = 355,400 BioProjects; ftp://ftp.ncbi.nlm.nih.gov/bioproject/summary.txt, accessed 1st May 2019), parsing it to extract a list of BioProject accessions with a data type of “transcriptome or gene expression” and an associated NCBI taxonomy ID of 9823 (*Sus scrofa*), 9825 (*Sus scrofa domesticus*), or one of 11 recognized subspecies: 291050, 309913, 309914, 375578, 310260, 310261, 375579, 415978, 490583, 1170810, and 2485929. Parsing this file only on the basis of exact species name would omit data due to inconsistent use: for example, BioProject PRJNA271310 records the species as *Sus Scrofa*, and PRJNA217840 as *Sus scrofa*.

We then used the Entrez Direct suite of utilities (https://www.ncbi.nlm.nih.gov/books/NBK179288/, accessed 1st May 2019) to associate each BioProject accession with a list of SRA sample and run accessions (a “RunInfo” file). RunInfo files were parsed to retain only those runs where “Platform” was “ILLUMINA,” “LibrarySource” was “TRANSCRIPTOMIC,” “LibraryStrategy” was “RNAseq,” “LibraryLayout” was “PAIRED,” “LibrarySelection” was either “cDNA” or “Inverse rRNA.” As an index of comparable quality and depth of sequencing, “avgLength” was ≥ 100 (i.e., a minimum average read length of 100 bp) and “spots” was ≥ 10 (i.e., approximating a minimum depth of 10 million reads).

The associated metadata were obtained using the R/Bioconductor package SRAdb (Zhu et al., 2013) to cross-reference each run accession with an SQL database of SRA metadata, SRAmetadb.sqlite (https://starbuck1.s3.amazonaws.com/sradb/SRAmetadb.sqlite.gz, accessed 29th April 2019). Metadata were not consistently recorded between authors, with naming conventions often loosely applied. Where possible, we obtained breed, sex, age, and tissue/cell type by parsing metadata lines to extract the values associated with (a) “breed” or “breed name” (else “strain”), (b) “sex,” “Sex,” or “gender,” (c) “age,” “developmental stage,” “developmental_stage,” “stage,” or “Stage,” and (d) “tissue,” “tissue type,” “tissue_type,” “organism part,” or “organism_part” (else “cell type” or “cell_type”). Only those samples with, at minimum, tissue/cell type recorded were incorporated into the expression atlas. The original metadata lines for each library, and the breed, sex, age and tissue/cell type extracted, are detailed in **Table S1**.

### Quantifying Gene Expression for the Atlas

For each library, expression was quantified using Kallisto v0.44.0 (Bray et al., 2016) as described in detail in previous studies on other species (Clark et al., 2017; Bush et al., 2018; Young et al., 2019). Kallisto quantifies expression at the transcript level, as transcripts per million (TPM), by building an index of k-mers from a set of reference transcripts and then “pseudo-aligning” reads to it, matching k-mers in the reads to k-mers in the index. Transcript-level TPM estimates were then summed to give gene-level TPM. For this purpose, an accurate set of reference transcripts was essential. To generate an accurate reference transcriptome, the pipeline runs Kallisto iteratively. The first pass utilises the largest available set of transcripts from the current reference transcriptome. Transcripts that are not detected in the first pass are removed from the reference to generate a modified reference.

To create the initial index, the set of Sscrofa11.1 protein-coding cDNAs, the batch release (ftp://ftp.ensembl.org/pub/release-96/fasta/sus_scrofa/cdna/Sus_scrofa.Sscrofa11.1.cdna.all.fa.gz, accessed 15th June 2019) from Ensembl v96 was parsed to retain only those transcripts with the ‘protein-coding’ biotype (n  =  45,788 transcripts, representing 22,340 genes). As Ensembl takes a conservative approach to annotation (Curwen et al., 2004), the set of 52,417 NCBI mRNA RefSeqs (representing 17,274 genes; ftp://ftp.ncbi.nlm.nih.gov/genomes/all/GCF/000/003/025/GCF_000003025.6_Sscrofa11.1/GCF_000003025.6_Sscrofa11.1_rna.fna.gz, accessed 15th June 2019) was parsed to supplement the index, including transcripts that had not already been assigned Ensembl transcript IDs and whose sequence was not already present in the Ensembl release (under any identifier). RefSeq mRNAs incorporate untranslated regions (UTRs) and so could encapsulate an Ensembl CDS. The trimmed UTRs from each mRNA were generated excluding all sequence outside the longest ORF. The method of integrating transcripts from both Ensembl and NCBI data sets is as previously described (Bush et al., 2018).

In total, the initial reference transcriptome comprised 98,205 transcripts, representing 28,254 genes. Using this reference, expression was quantified for 908 publicly archived paired-end Illumina RNAseq libraries, automatically obtained from the ENA (selected as described above) and detailed in **Table S1**. Prior to expression quantification, and for the purpose of minimising variation between samples, we randomly downsampled all libraries to 10 million reads, five times each, using seqtk v1.2 (https://github.com/lh3/seqtk, downloaded 4th June 2018). Expression level was then taken to be the median TPM across the five downsampled replicates.

The initial set of expression data was parsed to exclude genes for which the median TPM was less than 1 across all 908 samples (given the scope of the atlas, consistently unexpressed genes are either highly tissue-specific for a tissue not sampled, or erroneous models). 1,865 transcripts were removed to create a second Kallisto index (comprising 96,340 transcripts, representing 26,664 genes), and expression was requantified. For this purpose, the seqtk seeds used for downsampling were randomly assigned at run time.

As a final quality control test (described in (Bush et al., 2018)) we considered that in a correctly prepared RNAseq library, a minority of genes will produce the majority of reads and so the distribution of gene-level TPM estimates should comply, to a reasonable approximation, with Zipf’s law (i.e., that the probability of an observation is inversely proportional to its rank). On this basis, the exponent of a log-log plot of the reverse cumulative TPM per gene should not substantially deviate from an optimal value of −1. We identified 31 samples that were clear outliers and deviated by > 20% from this optimal value. These samples were excluded from further consideration. The final expression atlas details the median downsampled TPM per gene, BioProject, breed, and sex.

### Network Analysis and Functional Clustering of Atlas Samples

To examine the expression of genes across this wide range of tissues and cell types, the expression data were analyzed using the network analysis tool Graphia Professional (https://kajeka.com). The initial analysis used the values averaged by breed, sex, and BioProject for each tissue. Subsequent analyses used individual values for samples of liver and central nervous system. For each analysis, Pearson correlations (*r*) were calculated between all pairs of genes to produce a gene-to-gene correlation matrix and inversely between all sets of samples to produce a sample-to-sample correlation matrix.

Gene coexpression networks (GCNs) were generated from the matrices, where nodes represent either samples or genes and edges represent correlations between nodes above the correlation threshold indicated in the Results. For the sample-to-sample analyses (essentially analogous to a principal components analysis, PCA) an initial screen at the *r* value which entered all samples was performed, followed by subsequent analyses with higher *r* value which removed outliers and revealed more substructure in the networks. For each gene-to-gene analysis the *r* value was adjusted to retain the maximum number of genes with the minimum number of edges (**Figure S1**).

For the gene-to-gene networks, further analysis was performed to identify groups of highly connected genes within the overall topology of the network, using the Markov clustering algorithm (MCL) (van Dongen and Abreu-Goodger, 2012). The MCL is an algebraic bootstrapping process in which the number of clusters is not specified. A parameter called inflation effectively controls granularity. The choice of inflation value is empirical and is based in some measure on the predicted complexity of the data set. The chosen inflation value was 2.2 for the main atlas where we anticipated a large number of tissue-specific clusters, and 1.7 for the tissue/cell specific atlases to reduce the granularity (van Dongen and Abreu-Goodger, 2012). For the main atlas, only genes expressed at ≥ 10 TPM in at least one sample were included; for the tissue specific analysis genes expressed at ≥ 5 TPM in at least one sample were included. Gene ontology (GO) terms and pathways were derived from DAVID (https://david.ncifcrf.gov/; with *Sus scrofa* as the background) and GATHER (https://changlab.uth.tmc.edu/gather/). These approaches utilise different algorithms, methods of assessing significance and background data sets. Both were used to generate a consensus view of the functions of the clusters. Only the most significant results are reported (for DAVID, a Benjamini-Hochberg corrected P value of < 10^-2^; for GATHER a Bayes factor of ≥ 5).

## Results

### Samples in the Atlas

908 RNAseq libraries were obtained by the automated pipeline as described in Methods and used to create a global atlas of gene expression. For this purpose, expression across libraries was averaged by BioProject, tissue, breed and sex. This is equivalent to averaging across multiple biological replicates of the same tissue, sampled in the same lab, of the same sex and breed. This reduced the data set to 206 samples. For a separate analysis of liver and central nervous system to extract tissue-specific coexpression signatures, individual samples were used (102 samples for liver; 31 samples for central nervous system). Some of these individual samples were generated from RNAseq of pooled RNA.

### Network Analysis of the Pig Transcriptome

We used the network analysis program Graphia Professional (developed from BioLayout *Express* ^3D^) (Theocharidis et al., 2009) to create a network graph of the complete data set. Initially, we performed a sample-to-sample correlation to assess whether there were likely to be batch effects resulting in outlier samples. To include all 206 samples, it was necessary to use an *r* ≥ 0.38. In the resulting graph, all nodes (samples) were interconnected by a total of 14,243 edges, with liver samples in the same region and heart and muscle samples appropriately close. One hippocampus sample was separated from the other (adult) brain samples but this was a sample from foetal brain. Two samples, of ovary and gastrointestinal tract, had only a small number of connections. An image of the resulting network graph is shown in **Figure S2A**. To further explore the relationship between samples of the same organ system, we repeated the analysis at *r* ≥ 0.85. This produced a network graph which still retained 188 of the 206 samples, connected by 1,378 edges. Samples of related tissues analyzed in different Bioprojects generally clustered together. Liver samples were at the end of one arm of the network, close to adipose, while heart and muscle were on another arm.

Based on the sample-to-sample analysis, which did not identify any clear outliers or Bioproject-specific clusters (batch effects), we included all samples in the subsequent gene-to-gene analysis. The threshold correlation coefficient was chosen to maximise the number of nodes (genes) included while minimising the number of edges (correlations between them) (**Figure S1**). At the optimal correlation coefficient of *r* ≥ 0.70, the graph contained 19,861 nodes (genes) connected by 2,364,748 edges.

**Table 1** shows the expression patterns and biological processes associated with clusters of ≥40 nodes. There were distinct clusters that reflected the related functions of the genes contained within them: cell division (cluster 12), protein synthesis (clusters 2 and 37) and oxidative phosphorylation (cluster 20). There were also multiple tissue-specific clusters including transcripts that were expressed almost exclusively in one of the tissue types collected, including the testis (cluster 3), central nervous system (cluster 4), ovary (cluster 5), immune system (clusters 6, 16 and 39 (including macrophages)), lung (cluster 10), skeletal muscle (clusters 11 and 33), fibroblasts/mesenchyme (clusters 13 and 63), skin (cluster 15), thymus (cluster 17), adipose tissue (cluster 18), intestine (cluster 19), embryo inner cell mass (cluster 21), inner ear (cluster 23), epididymis (cluster 24), endometrium (cluster 25), peripheral blood (cluster 26), ileum (cluster 27), stem cells (cluster 28), and heart (cluster 31). Genes that were highly expressed in kidney (cluster 8) and liver (cluster 9) were also detected in the samples labelled “mixture of tissues” suggesting that these two tissues were a major component of the mixture and indicating that it is possible to deconvolute a heterogeneous sample using this approach.

**Table 1:** Gene expression clusters from pig tissues and cells. Clusters were generated at *r* ≥ 0.7 and MCL inflation value 2.2. Clusters of ≥40 nodes are shown.

Cluster number	Number of transcripts	Expression pattern	Class	Functional annotation
**1**	1,902	Digestive tract, CNS > immune > others	Housekeeping	Regulation of transcription, cell cycle
**2**	1,555	Ovary (some) > immune, kidney, adipose > liver, muscle, adipose	Protein synthesis	Protein biosynthesis, primary metabolism, ribosome, retrograde transport, endosome to Golgi
**3**	1,505	Testis only; many unannotated genes	Male reproductive	Sexual reproduction, male gamete generation, spermatogenesis, spermatid development, sperm motility
**4**	1,504	CNS	Central nervous system	Transmission of nerve impulse, synaptic transmission, cell communication, axon guidance, synapse organisation
**5**	787	Ovary (some) only; many unannotated genes	Female reproductive	Transcription, defence response
**6**	651	Spleen > lung, peripheral blood > lymph nodes, thymus, tonsil, macrophages	Immune system	Immune response, defence response, response to pest, pathogen or parasite, inflammatory response, innate immune response
**7**	560	Oocyte only	Female reproductive	Ubiquitin cycle, female gamete generation, nucleoplasm
**8**	502	Kidney >> liver, ovary (two), mixture of tissues	Kidney	Excretion, organic acid metabolism, sodium ion transport, cell communication
**9**	445	Liver > mixture of tissues	Liver	Organic acid metabolism
**10**	321	Lung >>> adipose, mixture of tissues	Respiratory system	Morphogenesis, organ development, regulation of liquid surface tension, angiogenesis, patterning of blood vessels, heart development
**11**	319	Muscle >> heart, mixture of tissues	Muscular system	Muscle contraction, muscle development, myofibril assembly, glucose/hexose/carbohydrate metabolism, glycolysis, gluconeogenesis
**12**	279	Fetal thymus, cell lines > immune, ovary > digestive tract, adipose, kidney	Cell division	Cell cycle, cell proliferation, mitosis, DNA replication, microtubule based movement, G1/S transition of mitotic cell cycle
**13**	264	Mesenchymal cells > adipose, muscle (some), ovary, lung	Connective tissue	Development, phosphate transport, cell communication, skeletal development, extracellular matrix, collagen triple helix repeat
**14**	262	Stem cells > brain > immune, ovary, adipose, digestive tract	Development	RNA processing, organelle organisation and biogenesis, mRNA splicing *via* spliceosome, nucleosome assembly
**15**	274	Ear >>> adipose (some)	Integumentary system	Epidermis development, ectoderm development, catabolism, keratinocyte differentiation
**16**	187	BMDM >> alveolar macrophages, immune	Immune system	Immune response, defence response, response to pest, pathogen or parasite, response to wounding, inflammatory response, lysosome, monocyte chemotaxis
**17**	181	Fetal thymus >>> immune system, blood	Immune system	Defence response, immune response, T-cell activation, lymphocyte differentiation, hemopoiesis,
**18**	170	Adipose only	Integumentary system	Lipid metabolism, energy derivation by oxidation of organic compounds, cell-matrix adhesion, regulation of lipolysis in adipocytes
**19**	169	Large intestine > small intestine	Digestive system	Mineral absorption, ion transport
**20**	156	Heart > muscle > kidney, mixture of tissues > digestive tract > liver, lung, immune	Mitochondria	Oxidative phosphorylation, electron transport, ATP synthesis coupled electron transport, tricarboxylic acid cycle, respiratory chain, aerobic respiration
**21**	154	Ovary (three only); many unannotated genes	Female reproductive	Steroid metabolism, lipid metabolism, cholesterol metabolic process, ovarian steroidogenesis, reproductive physiological process
**22**	147	Inner cell mass from day 7-8 embryos only	Development	Cell communication, membrane lipid catabolism,
**23**	151	Inner ear stria vascularis only	Integumentary system	Sensory perception of mechanical stimulus, perception of sound, ion transport, morphogenesis, skeletal development, signal, collagen triple helix repeat
**24**	131	Epididymis	Male reproductive	Defensin, defence response to bacterium, innate immune response,
**25**	127	Uterine endometrium > chorion	Female reproductive	Protein catabolic process, glycosphingolipid metabolism
**26**	125	Peripheral blood only	Immune system	Response to biotic stimulus, defence response, response to pest, pathogen or parasite, immune response, response to wounding, osteoclast differentiation
**27**	116	Ileum >>> other digestive tract	Digestive tract	Nucleotide transport, digestion, bile secretion
**28**	157	Stem cells >> iPSC; mainly unannotated genes	Development	Pattern specification, axis specification
**29**	112	Hippocampus (fetal) > oocyte > random samples; mainly unannotated genes	Olfactory receptors	Perception of smell, sensory perception, neurophysiological process
**30**	107	Fibroblasts > somatic cells	Integumentary system	Neuromuscular junction, heparin binding, extracellular exosome, morphogenesis, development, growth
**31**	104	Heart >> muscle > mixture of tissues	Cardiovascular system	Cardiac muscle contraction, ventricular cardiac muscle tissue morphogenesis, circulation, muscle development, regulation of heart contraction rate
**32**	85	Duodenum >> ileum, caecum, colon	Digestive tract	Digestion, cobalt/metal ion transport, extracellular region
**33**	86	Longissimus dorsi muscle (two only) >> other muscle, heart, chorion	Muscular system	Muscle development, muscle contraction, myogenesis
**34**	74	Pituitary gland only; many unannotated genes	Endocrine system	Sex differentiation, pregnancy, neuroactive ligand-receptor interaction, hormone, extracellular region, hormone mediated signalling pathway
**35**	73	Embryo, inner cell mass > ovary (most), fetal thymus, random others	Development	RNA metabolism, RNA processing, protein folding, chaperonin-containing T complex, positive regulation of protein localisation of Cajal body, binding of sperm to zona pellucida, metabolism, toxin transport
**36**	72	Hippocampal formation, amygdala (one) >> other CNS	Nervous system	Locomotory behaviour, neuropeptide signalling pathway, adenylate cyclase-activating dopamine receptor signalling pathway, signalling pathway, cell communication, synaptic transmission
**37**	72	Digestive tract, spleen, ovary > adipose, lung, blood > muscle, heart, liver, kidney	Pathway	Protein biosynthesis, translation, ribosomal assembly, metabolism
**38**	70	Stomach >>> fibroblasts, somatic cells	Digestive tract	Smooth muscle contraction, muscle development, cGMP-PKG signalling pathway
**39**	69	Alveolar macrophages > BMDMs > lung	Immune system	Defence response, immune response, response to (external) biotic stimulus, phagosome
**40**	66	Penis >> tonsil >> alveolar macrophages	Immune system	Inflammatory response, response to wounding, immune response, calcium ion binding
**41**	64	Primordia of developing teeth only	Development	Neurogenesis, organogenesis, morphogenesis, homeodomain
**42**	64	Kidney cell line	Kidney	Integrin mediated signalling pathway, cell-matrix adhesion
**43**	58	iPSC (one) > other stem cells	Development	Regulation of metabolism, regulation of transcription, development, signalling pathways regulating pluripotency of stem cells, neurogenesis
**44**	55	Immune, digestive tract	Immune system	Defence response, response to biotic stimulus, immune response, external side of plasma membrane
**45**	53	Mixture of multiple tissues (one) >> random other samples; many unannotated genes	Mixture	Cortical cytoskeleton
**46**	53	iPSC >> CNS, digestive tract	Development	Cellular metabolism
**47**	51	BMDM after LPS, some spleen, some lung > blood, some adipose, some ovary > digestive tract	Immune system	Response to biotic stimulus, immune response, defence response, defence response to virus, negative regulation of viral genome replication, ubiquitin cycle, ISG15 protein conjugation
**48**	50	Oocyte > digestive tract, CNS, kidney, lung, spleen, immune, ovary > liver, heart, muscle, adipose	Development	Ubiquitin cycle
**49**	50	Digestive tract, forebrain, tonsil, lymph nodes, spleen (one) > ovary (two), male reproductive	Digestive tract	Nucleosome assembly, chromatin assembly or disassembly, DNA packaging
**50**	49	Duodenum > other intestine, spleen, alveolar macrophages	Digestive tract	Humoral immune response, response to pest, pathogen or parasite, antimicrobial humoral response, inerleukin-10 biosynthesis, interleukin-4 biosynthesis, interleukin-13 biosynthesis
**51**	48	Ovary (most), CNS	Female reproductive	Transport, localisation
**52**	47	Kidney only, one sample very high	Kidney	Perception of small, sensory perception
**53**	47	Endometrium of pregnancy >> uterine endometrium	Female reproductive	Di-, tri-valent inorganic cation transporter
**54**	44	Adipose, lung, ovary, spleen > digestive tract, macrophages > heart	Connective tissue	*No significant annotation*
**55**	43	Ovary (one) >> ovary (two); many unannotated genes	Female reproductive	Hormone biosynthesis
**56**	42	Testis > CNS, digestive tract > other male reproductive	Male reproductive	*No significant annotation*
**57**	41	Pituitary gland only; many unannotated genes	Endocrine system	Secretory granule
**58**	41	Inner cell mass >> adipose, kidney, lung, spleen, BMDM, ovary	Development	*No significant annotation*
**59**	41	Liver (one); mainly unannotated genes	Liver	*No significant annotation*

As observed in other atlas projects (Hume et al., 2010; Freeman et al., 2012; FANTOM Consortium et al., 2014; Clark et al., 2017; Bush et al., 2018; Young et al., 2019) tissue-specific coregulated clusters contained the transcription factors that likely drive coexpression. For example, the thymus/T cell cluster (cluster 17) contains the transcription factors *LEF1* and *TCF7* which are known T lymphocyte regulators and the inner cell mass cluster (cluster 21) contains *NANOG* and *PRDM14* which are involved in cell pluripotency. **Table S2** (Cluster profiles and gene lists sheet) provides lists of the genes in each cluster and associated histograms show the average expression profile of all clusters containing 10 or more genes.

**Figure 1:**
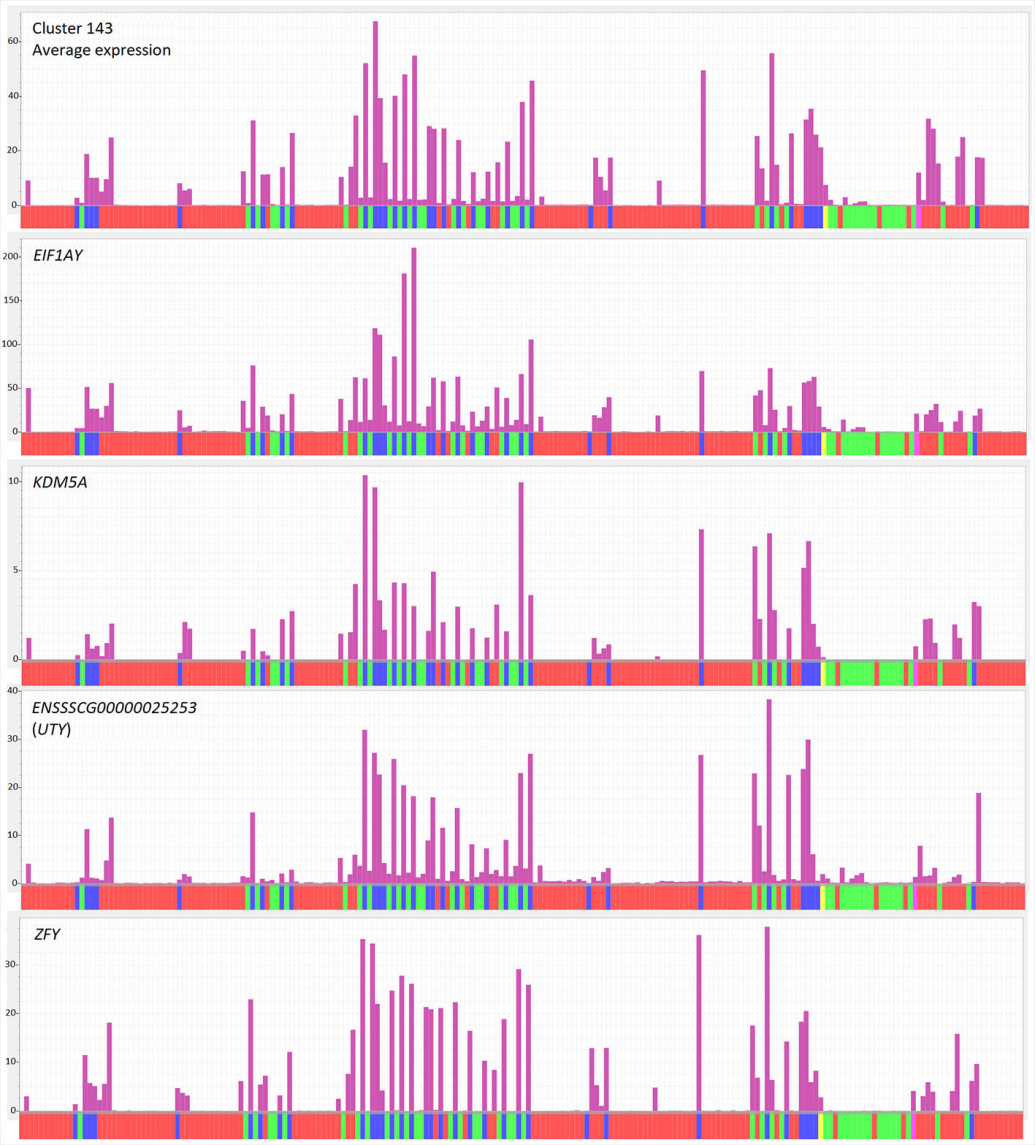
Expression of Y chromosome genes across tissues in the atlas. Top panel shows average expression of cluster 143, which contains genes mapped to the Y chromosome. Remaining panels show expression of Y chromosome genes from cluster 143. Y axis shows normalized expression levels (TPM) derived from Kallisto (see Methods). Each bar on the X axis represents a sample in the analysis, averaged for breed, sex and BioProject. The order of samples is as for **Table S2**. Red indicates sex unknown, green indicates female, blue indicates male and yellow indicates pooled samples of mixed/unknown sex.

The transcriptomic data generated for the Sscrofa11.1 pig genome (BioProject PRJEB19386) (Warr et al., 2019) included males and females for most tissues. In other data sets, the sex is not always provided with the metadata. This issue can be addressed for most data sets by examining the expression of transcripts transcribed from the Y chromosome which are necessarily expressed only by males. For instance, cluster 143, with seven genes and 11 transcripts, only contained Y-specific protein-coding genes (*EIF1AY*, *EIF2S3Y, DDX3Y, KDM5D, TXLNGY, USP9Y*, *UTY*, *ZFY*) (**Figure 1**; gene list in **Table S2**, Cluster profiles and gene lists sheet). The coordinated expression of these transcripts can effectively be used to sex each sample. The fact that this cluster does not contain transcripts from the autosomes indicates that there are no other transcripts that are robustly expressed in males and undetectable in females.

Each of the clusters contains multiple transcripts that are currently represented in Ensembl by placeholder “ENSSSCG” or “LOC” IDs (**Table S2**). As noted previously in multiple species (Hume et al., 2010; Freeman et al., 2012; FANTOM Consortium et al., 2014; Carpanini et al., 2017; Clark et al., 2017; Bush et al., 2018) poorly annotated transcripts were more prevalent in the clusters of genes that are most widely expressed, reflecting the historical emphasis on tissue-specific genes in functional genomics (**Table S2**, ENSSSCG and LOC genes sheet). In many cases, the likely identity is actually evident from orthology analysis available in Ensembl but has not yet been adopted as an official gene name in Sscrofa11.1. **Table S2** includes provisional annotations from GeneCards or NCBI for each of these transcripts where available. The clear potential of the clusters to support prospective annotation and orthology assignments for those transcripts that lack current informative annotation is evident from the high levels of GO enrichment in **Table 1**.

It was not our intention to annotate all of the transcripts. This is an activity that requires a community-based effort and manual annotation (Dawson et al., 2013; Dawson et al., 2017). In the specific case of the brain/CNS-enriched cluster (cluster 4) LOC IDs have clearly been assigned to candidate members of multigene families, notably the large zinc finger family in which strict orthology relationships are difficult to determine. We examined each of the novel ENSSSCG genes (that is, unannotated genes) within cluster 4 individually (**Table S2**, ENSSSCG genes cluster 4 sheet). In each case, we used NCBI BLAST (August, 2019) to identify candidate DNA orthologs in the human genome. As shown in **Table S2** the large majority of ENSSSCG genes annotated as “no class” have unique identity to exons (including alternative internal exons, 3' and 5' UTRs) of known human genes. In each case, we checked whether the corresponding gene is also expressed and enriched in human CNS tissue using combined GTEX, FANTOM5, and HPA data (https://www.proteinatlas.org). In many cases, the corresponding annotated pig transcript is also present in cluster 4 (i.e. the novel ENSSSCG gene is coexpressed with an annotated gene that corresponds with known human transcript in the expected genomic location based on conservation of synteny). For example, *LLRC7*, which encodes the excitatory synaptic protein densin-180 (Wang et al., 2017) is expressed in a CNS-specific manner in humans. The pig DNA sequences corresponding to human *LLRC7* were annotated as three separate genes in Suscrofa11.1 (Ensembl release 90) all of which are contained within the same genomic region and with the same transcriptional orientation. These transcripts are merged in a single gene, *LRRC7* (ENSSSCG00070015233) in the Ensembl annotation of the assembly of a second pig genome, USMARCv1.0, and have also now been merged in revised annotation of Sscrofa11.1 (Ensembl release 98) (Warr et al., 2019). We conclude that many current ENSSSCG transcripts will be recognized as alternative transcripts of known genes with improved annotation based upon full length cDNA transcripts.

### The Transcriptome of Pig Macrophages

Previous studies of the pig transcriptome using microarrays identified transcripts that were enriched in the macrophages of the lung and in subsets of blood monocytes as well as transcripts that were inducible by bacterial lipopolysaccharide (LPS). (Freeman et al., 2012; Kapetanovic et al., 2012; Schroder et al., 2012; Fairbairn et al., 2013; Kapetanovic et al., 2013). As discussed above, macrophages in other species adapt to tissue-specific niches to perform specific functions. Several clusters within the atlas are enriched for monocyte-macrophage expressed transcripts. By inference other transcripts within those clusters form part of the so-called immunome (Dawson et al., 2013) and are likely to be involved in innate immunity. Cluster 6 contains macrophage-specific transcription factors (*IRF8, MAFB*, *SPI1*), the lineage-specific growth factor receptor, *CSF1R,* widely used macrophage markers including *CD68, SIRPA, ITGAM,* and *SIGLEC1,* and class II MHC genes. The average expression is high in the isolated macrophages and in macrophage-rich tissues, notably spleen and lung. This large cluster represents a generic monocyte-macrophage signature for the pig.

Consistent with the earlier findings, there is a cluster of transcripts (cluster 39) that are highly expressed in alveolar macrophages (AM) relative to BMDM. Because macrophages are a major cell population in the lung, high expression of these genes is also evident in total lung mRNA. The AM-enriched cluster includes CD163, identified as an essential fusion receptor for the major pig respiratory pathogen PRRSV (porcine reproductive and respiratory syndrome virus) (Burkard et al., 2018) and the receptor for CSF2 (*CSF2RA* gene), which is required for lung macrophage homeostasis in humans and mice (Trapnell et al., 2019). AM are enriched for expression of lectin-like receptors (*MRC1, CLEC7A, CLEC2B*) and *CYP7A1*. Transcripts encoding other macrophage-expressed surface markers (*ADGRE1, CH3L1*) and endocytic receptors (*CLEC14A, CLEC1A, HAVCR2, TIMD4, VSIG2, VSIG4*) that are even more lung-restricted are found within the lung-specific cluster 10. By contrast, as noted previously, the lung macrophage-enriched transcripts are barely detected in the wall of the gut which contains an abundant macrophage population. We conclude that lung macrophages are adapted specifically to phagocytose inhaled pathogens. A reciprocal cluster, cluster 16, contains transcripts that are also monocyte-macrophage-enriched but more highly expressed in BMDM than in AM. By contrast to cluster 10, these genes are detected in the intestinal samples. In the overall atlas above (**Table S2**) there is an additional small liver-specific signature, cluster 183, that includes *CD5L*, *CLEC4F,* and genes involved in iron recycling (*SLC40A1, HEBP1*), each associated with the macrophages of the liver (Kupffer cells) in mice (Scott et al., 2018).

This cluster analysis, based upon RNAseq data, confirms the major clusters of LPS-inducible genes previously analyzed in the pig using microarrays (Freeman et al., 2012; Kapetanovic et al., 2012; Schroder et al., 2012; Fairbairn et al., 2013; Kapetanovic et al., 2013). On average, transcripts in clusters 16 were strongly induced by LPS in BMDM with lower expression in other sites such as spleen, lung and AM. The cluster includes the inflammatory cytokine *IL1B* and several interferon-inducible transcripts. Although the primary RNAseq data confirm their regulation by LPS, cluster 16 does not include transcripts such as *CCL20, CYP27B1,* and *IDO1* which are LPS-inducible in human and pig macrophages, but not in mice (Schroder et al., 2012). These transcripts are expressed at much higher levels in other tissues and fall with different clusters. The data also confirm that pig macrophages do not induce nitric oxide synthase (*NOS2*) in response to LPS (Young et al., 2018). In the pig, as in human (see data on https://www.proteinatlas.org), *NOS2* is expressed specifically in the gut.

### The Transcriptome of the Pig Liver

The downloaded data sets included 102 individual RNAseq libraries of liver, from various ages, sexes and breeds derived from multiple BioProjects that met the QC and filtering criteria. Liver gene expression is regulated in response to numerous physiological stimuli. Aside from hepatic parenchymal cells, the liver contains several nonparenchymal populations. To identify coregulated clusters within the liver transcriptome, we analyzed the liver samples separately using the same GCN approach used for the overall atlas. When examining individual samples from the same tissue, the method exploits variability in gene expression between samples generated by differences in breed, sex, age, sampling (biopsy versus necropsy), and other factors, which among other outcomes, can enable deconvolution of the expression signals associated with specific cell types. In principle, if there was significant heterogeneity in metabolic state or development among the liver samples, a gene-to-gene clustering might reveal sets of genes associated with portal versus centrilobular regions of liver lobules. Centrilobular genes were found to be downregulated in liver of pigs treated with macrophage colony-stimulating factor (CSF1) (Sauter et al., 2016) as also seen in regenerating liver in other species. **Table 2** shows the GO term enrichment for the largest clusters identified using the liver samples, generated at Pearson correlation threshold of 0.75 (**Figure S2B**) and **Table S3** contains lists of genes within these clusters.

**Table 2:** Gene expression clusters from pig liver. Clusters were generated at *r* ≥ 0.75 and Markov clustering algorithm (MCL) inflation value 1.7. Clusters of ≥40 nodes are shown.

Cluster number	Number of nodes	GO term enrichment
**1**	3648	Protein modification, primary metabolism, protein transport, protein localization, G-protein coupled receptor protein signalling
**2**	1951	Biosynthesis, macromolecule metabolism, neurophysiological process, response to external stimulus, extracellular exosome, translation, mitochondrion
**3**	753	Cytoplasm organization and biogenesis, ribosome biogenesis and assembly, RNA processing and metabolism, RNA splicing, cellular physiological process, poly(A) RNA binding, nucleolus
**4**	599	*No significant GO term enrichment*
**5**	325	Phosphate transport, development, regulation of cellular process, anion transport, morphogenesis, cell adhesion, extracellular exosome, extracellular matrix, angiogenesis,
**6**	248	Organic acid metabolism, lipid metabolism, amine metabolism, steroid metabolism, mitochondrion, fatty acid beta oxidation using acyl-CoA dehydrogenase, lipid homeostasis
**7**	169	Immune response, defence response, response to biotic stimulus, response to wounding, inflammatory response, positive regulation of T cell proliferation
**8**	159	Signal transduction, cell communication, immune cell migration, integrin mediated signalling pathway, regulation of cell shape, leukocyte cell-cell adhesion
**9**	157	Protein transport, establishment of protein localisation, secretory pathway, Golgi vesicle transport, ER-associated protein catabolism, protein folding, endoplasmic reticulum chaperone complex, response to endoplasmic reticulum stress
**10**	120	*No significant GO term enrichment*
**11**	105	Electron transport, generation of precursor metabolites and energy, amine metabolism, regulation of blood coagulation, extracellular exosome
**12**	100	Response to unfolded protein, protein folding, response to stress
**13**	93	*No significant GO term enrichment*
**14**	89	Response to biotic stimulus, immune response, defence response, ubiquitin cycle, defence response to virus, negative regulation of viral genome replication, ISG15-protein conjugation
**15**	89	*No significant GO term enrichment*
**16**	65	Generation of precursor metabolites and energy, electron transport, oxidative phosphorylation, ATP coupled electron transport, metabolism, cytochrome C oxidase activity, NADH dehydrogenase (ubiquinone) activity
**17**	60	Carboxylic acid metabolism, pigment metabolism, anti-inflammatory response, extracellular exosome, blood microparticle, extracellular matrix
**18**	60	Transition metal ion transport, oxidation-reduction process
**19**	47	*No significant GO term enrichment*
**20**	47	Fatty acid metabolism pathway, mitochondrial inner membrane
**21**	43	Structural constituent of ribosome, translation, cytosolic large/small ribosomal subunit, focal adhesion, nucleolus
**22**	40	*No significant GO term enrichment*

In rodents, there is a set of transcripts that is expressed in the liver in a sex-specific manner in part under the influence of growth hormone (Conforto et al., 2012; Lau-Corona et al., 2017). The male and female-specific liver transcriptomes are regulated by differential expression of specific transcription factors, CUX2 and ONECUT2 in females and BCL6 in males. A meta-analysis of human liver transcriptome data (Zhang et al., 2011) revealed some bias in gene expression between males and females but the differences were considerably smaller than reported in rodents, notably associated with the sex chromosomes and predominantly female-biased. The complete set of liver samples is shown in **Table S3** with the male and female samples indicated where possible based upon metadata and expression of Y chromosome-specific transcripts. The majority of the liver samples available in the pig are male. In a sample-to-sample analysis, most definitive female samples (identified based on absence of Y chromosome-specific transcripts) separated from the male group but in the gene-to-gene analysis there was no cluster of transcripts that were enriched or depleted in the female livers, other than those encoded by genes on the Y chromosome. Given the rigorous genetic selection of commercial pigs for rapid growth, early puberty and lean muscle, it may be that the differential gene expression driven by growth hormone in other species is masked by high levels of growth hormone in both pig sexes.

The two largest clusters in the liver analysis reflect variation between the available BioProjects; mainly between a large study of a model of hemorrhagic shock, which extracted RNA from liver biopsy material (Determan et al., 2014), and all of the other liver data sets. The hemorrhagic shock study includes individual time courses of postshock recovery and examines the effect of starvation versus carbohydrate prefeeding. Cluster 1, comprising genes which are expressed somewhat lower on average in samples from this study, contains the hepatocyte-specific transcription factors (e.g., *HNF4G, HNF1A*) and numerous liver-enriched transcripts (see below). The reciprocal large clusters (clusters 2 and 6) that are elevated in the shock study include multiple heat shock proteins and genes encoding metabolic enzymes (assigned GO terms including lipid metabolism, amine metabolism, and fatty acid beta oxidation; **Table 2**) that may be upregulated either by the trauma or the dietary interventions.

Smaller clusters are more clearly enriched for function as evident from GO term enrichment. Cluster 11 contains *CYP2E1* and multiple other transcripts encoding xenobiotic-metabolising enzymes that are normally enriched in centrilobular regions. Many of these centrilobular-enriched genes were also downregulated by CSF1 treatment in pig liver (Sauter et al., 2016). Cluster 12 contains further transcripts encoding heat shock proteins and other stress related genes and is enriched for the GO terms “response to unfolded proteins” and “response to stress.” Both of these clusters appear to be coordinately regulated in the shock model (Determan et al., 2014) (the profiles show an increase with time in each of the animals in the study). Cluster 20 contains further genes associated with lipid metabolism and cluster 21 numerous ribosome-associated transcripts. Cluster 14 is enriched for the GO terms “immune response” and “defence response to virus” and contains the interferon-regulated transcription factors, *IRF7, IRF9,* and *STAT1* and many known interferon target genes (*MX1, IFIT5, RSAD2*). This cluster presumably reflects some variation in the health status of the animals in each of the projects.

Other smaller clusters are likely to indicate variable contributions from the major nonparenchymal cell types to the total mRNA pool of each liver sample. For example, cluster 5 (enriched for GO terms including “cell adhesion” and “extracellular matrix”) contains mesenchyme-associated transcripts encoding multiple collagens (e.g., *COL1A1*) extracellular matrix proteins (e.g., *BGN, DCN, FBLN2*) and growth factors/receptors (*BMP4, FGFR1, PDGFB, PDGFRB*) (Hume et al., 2010; Summers et al., 2010) that likely reflect variation in the relative abundance of hepatic stellate cells in different samples.

Cluster 7 (enriched for the GO term “immune response”) is a generic blood leukocyte cluster that contains the pan-leukocyte markers *PTPRC* (CD45). This cluster is driven by one sample with high expression of the cluster genes, which presumably had a larger blood contamination than other samples. Cluster 8 (which is also enriched for immune-associated GO terms) contains monocyte-associated transcripts (e.g., *CSF1R, CD68, IRF5*), whereas cluster 56 contains four known markers of resident liver macrophages (*CD32, CD163, TIMD4, VSIG4*) that were selectively expanded in the pig liver in response to CSF1 treatment (Sauter et al., 2016). This cluster also contains *ADGRE1* (which in mouse encodes the widely used macrophage marker F4/80 antigen) and related *EMR4* (*ADGRE4*). We recently produced a monoclonal antibody against pig ADGRE1 and demonstrated binding to Kupffer cells (Waddell et al., 2018) although as discussed below, expression was highest on alveolar macrophages.

### The Transcriptome of the Pig Brain

Cluster 4 of the whole atlas (**Table S2**) contains genes that are clearly enriched in the CNS. It includes a small subset of the signatures of specific cell types in brain, including brain macrophages (microglia; e.g., *CX3CR1, P2RY12*) and oligodendrocytes (*OLIG1, OMG*) but does not segregate region-specific function, nor identify transcripts that may have brain-specific functions. Studies in mice, rats, and humans have extracted a core microglial signature by network analysis of multiple brain gene expression profiles, alongside signatures of multiple cell types and region-specific functions (Carpanini et al., 2017; Pridans et al., 2018; Patir et al., 2019). To dissect coexpression profiles in the CNS, we performed a separate network analysis of all of the available CNS samples individually. Samples from different regions were generally separated in the sample-to-sample analysis (**Figure S2C**). **Table 3** lists the major clusters and associated GO terms generated at a correlation coefficient of 0.85 and **Table S4** shows the genes in the clusters and their expression profiles. This analysis segregates the CNS-associated transcriptome to some extent based upon region. The average expression of transcripts in cluster 1 (the largest cluster) of this CNS analysis is highest in the cortex and increased with time in a developmental time course. Transcripts in clusters 2 and 14 are both strongly enriched in pituitary, although the two clusters differ in relative expression between the males and females. Cluster 2 includes transcripts encoding four major pituitary hormones (*FSH, GH1, LHB, TSHB*) and the receptors that regulate secretion (e.g. *ESR1, GHR, GNRHR, GSHR, TRHR*). Many transcriptional regulators of pituitary development are also contained with cluster 2, including *ISL1, GATA2, PITX1, PITX2, POU1F1, PROP1, SIX3,* and *SIX6* (Charles et al., 2006; Castinetti et al., 2015; Xie et al., 2015; Sobrier et al., 2016). Cluster 14 contains transcripts encoding the other major pituitary hormone, *POMC*, regulatory receptors *GHRHR* and *CASR* (Mamillapalli and Wysolmerski, 2010) and the key transcriptional regulator of *POMC, PAX7* (Drouin, 2016). Cluster 2 was enriched for GO terms associated with extracellular exosome and oligosaccharyl transferase; cluster 14 was not significantly enriched for GO terms. Based upon their strong association, many of the transcripts within these two clusters are likely to contribute to pituitary development or function.

**Table 3:** Gene expression clusters from pig central nervous system. Clusters were generated at *r* ≥ 0.85 and Markov clustering algorithm (MCL) inflation value 1.7. Clusters of ≥40 nodes are shown.

Cluster number	Number of nodes	Expression pattern	GO term enrichment
**1**	2,175	PRNJA40675 > developing cortex (decreases with age)	Nucleobase, nucleoside, nucleotide and nucleic acid metabolism, organismal physiological process, transcription, primary metabolism, response to external stimulus, defence response, immune response, perception of smell, biopolymer metabolism, poly(A) RNA binding, metal ion binding
**2**	1,118	Female pituitary > male pituitary > all others	Extracellular exosome, oligosaccharyl transferase complex
**3**	1,113	Pituitary > brain regions > developing cortex, hypothalamus	Immune response, defence response, response to biotic stimulus, T-cell activation, organismal physiological process, lymphocyte activation, response to wounding
**4**	1,054	Forebrain > cortex (decreasing with age) > others	Cell cycle, cell proliferation, DNA metabolism, M phase of mitotic cell cycle, nuclear division, mitosis, signal transduction, primary metabolism, kinetochore, nuclear chromatin, translation, DNA replication-dependent nucleosome assembly
**5**	657	Hypothalamus > brain regions > frontal and occipital cortex > developing cortex (decreases with age)	Nucleobase, nucleoside, nucleotide and nucleic acid metabolism, glycolysis, intracellular signalling cascade, catabolism,
**6**	504	Female pituitary, hypothalamus > male pituitary, brain regions	G-protein coupled receptor protein signalling metabolism, cell surface receptor linked signal transduction
**7**	483	Brain regions including hypothalamus > hippocampal formation, cortex > pituitary, “brain”, developing cortex, hippocampus	Cell communication, nucleobase, nucleoside, nucleotide and nucleic acid metabolism, signal transduction, response to wounding, myelin sheath
**8**	345	Hypothalamus	Regulation of cellular process, immunity
**9**	298	Brain regions > pituitary, “brain”, hippocampus	Transport, localisation, monovalent cation transport, synaptic transmission, transmission of nerve impulse, cell-cell signalling, vesicle mediated transport, myelin sheath, synapse, SNARE complex
**10**	231	Hippocampus, mainly ENSSSCG and LOC	Defence response, immune response, response to biotic stimulus
**11**	174	General, variable	Electron transport, generation of precursor metabolites and energy, mitochondrial electron transport, NADH to ubiquinone, ATP synthesis coupled electron transport, mitochondrial respiratory chain complex I
**12**	144	Hypothalamus > “brain” > others	*No significant GO term enrichment*
**13**	102	One sample of cortex (SRS1027244) > one sample of brain (SRS2520758) > all others	Sexual reproduction, male gamete generation, spermatogenesis, anion transport
**14**	88	Male pituitary > female pituitary, all others very low	*No significant GO term enrichment*
**15**	82	Hypothalamus (SRS497445) > > Hypothalamus (SRS497448) > all others	Cell-cell signalling, synaptic transmission, neuropeptide signalling pathway
**16**	74	Developing cortex, forebrain, brainstem >> all others	Coenzyme/cofactor biosynthesis, energy coupled proton transport, down electrochemical gradient, ATP biosynthesis, mitochondrion
**17**	73	Developing cortex decreasing with age, “brain” and brain regions moderate, hypothalamus low	Protein modification
**18**	71	Female pituitary, forebrain > other brain regions > hypothalamus	Histidine family amino acid catabolism, histidine metabolism
**19**	71	Brain, brain regions > hypothalamus, forebrain, hippocampal formation, hippocampus	*No significant GO term enrichment*
**20**	68	Cortex (SRS1027246) >> other cortex, “brain” > other brain regions	Development, regulation of biological process
**21**	63	Brain, some cortex, corpus callosum, brain stem (SRS1027238) > others	Cell-cell signalling, ion transport, synaptic transmission, transmission of nerve impulse, postsynaptic membrane
**22**	62	Amygdala, hippocampal formation > cortex (SRS1027243) > others	G-protein coupled receptor protein signalling, cell communication, signal transduction, synaptic transmission, transmission of nerve impulse, dopaminergic synaptic transmission
**23**	56	Low in pituitary, hippocampus, forebrain	Transport, localisation, chloride transport, secretory pathway, small GTPase mediated signal transduction
**24**	52	Hypothalamus, “brain” > pituitary, amygdala > other brain regions	*No significant GO term enrichment*
**25**	47	Forebrain, pituitary	*No significant GO term enrichment*
**26**	47	Hypothalamus, “brain”, pituitary > other brain regions	*No significant GO term enrichment*
**27**	44	Brainstem, cortex > “brain” > other regions	*No significant GO term enrichment*
**28**	44	Forebrain, hypothalamus, pituitary > other regions	*No significant GO term enrichment*
**29**	43	Cortex (one sample) > pituitary > other regions	*No significant GO term enrichment*
**30**	40	Pituitary (one sample) > all other samples	*No significant GO term enrichment*

Pituitary function is regulated by the hypothalamus which, together with the adrenal gland, forms the HPA axis. Clusters 8 and 15 contain transcripts that are strongly expressed in the hypothalamus. Cluster 8 is enriched for immune-associated transcripts (and GO terms pertaining to immunity) and includes *IL1A.* Cluster 15 includes transcripts encoding the hypothalamic hormones *OXT* and *GHRH,* the regulator of stress hormone release *SCGN* (Romanov et al., 2015) and the transcription factor *LHX1* (Bedont et al., 2017). Several other clusters show idiosyncratic average profiles across brain regions, including upregulation or downregulation in the developmental time course of cortex. Some clusters, such as clusters 21 and 22, contain multiple receptors associated with neuronal responses to specific neurotransmitters (dopamine, GABA, glutamate) and may reflect neuronal cell type-specific clusters. Others are clearly associated with general biological functions that vary between brain regions or during development, for example the cell cycle (cluster 4), oxidative (cluster 11) and glycolytic (cluster 5) metabolism, and intracellular transport processes (cluster 9). By contrast to studies in other species, the network analysis did not segregate a clear signature of the major glial cell types, microglia, oligodendrocytes and astrocytes. Microglia-associated transcriptional markers (Carpanini et al., 2017; Pridans et al., 2018; Patir et al., 2019) are found within clusters 5 (*AIF1, C1QA*, *CD68, LAPTM5, TMEM119* (=*ENSCG00000027124*)) and 7 (e.g., *C5AR1, CSF1R, CX3CR1*, *GPR34, IRF8*). Cluster 7 also contains numerous oligodendrocyte-associated genes (*MBP, OLIG1*), perhaps reflecting the association between microglia and myelination during development [see (Pridans et al., 2018)]. Interestingly, the two key microglial growth regulators, *IL34* and *CSF1*, which show region-specific expression in mice and humans (Chitu and Stanley, 2017) are within separate clusters 5 and 7 respectively. IL34 was not brain-specific in the main atlas. It controls the differentiation of microglia and of Langerhans cells of the skin and does not cluster because it is expressed from different promoters in the two locations in mice and humans (FANTOM Consortium et al., 2014).

## Discussion

### Metadata Parsing Limits the Automatic Generation of Expression Atlases

For this study, we sought to reuse as much public RNAseq data for pigs as possible. By sampling a large transcriptional space, we considered that a more robust all-against-all correlation matrix would be created, allowing greater insight into gene coexpression patterns.

While the development of alignment-free methods of expression quantification greatly facilitates this aim, they have also repositioned the rate-limiting step of an RNAseq analysis pipeline. For example, decompressing NCBI.sra files (a binary representation of fastq) can be far slower than analysing the associated fastq data with Kallisto, referencing an index transcriptome.

The most time-consuming step in this study was selecting samples for inclusion in the expression atlas, as this requires manual review. Metadata were not always available for each sample or, if available, the metadata were not always complete. Common problems included typographical errors (for example, the samples in BioProject PRJNA340232 are “longissimus muslce”), missing units (the age of run SRR5110497 is “1”), and misplaced entries (the developmental stage of sample SRS986719 is “lean-type pig breeds”): products of human error that hinder an automated parser.

One major problem with the automated pipeline relates to the paucity of metadata for many samples. Often, the information on breed, sex, or age was available by accessing the BioProject or the publications cited therein directly, but this could not be captured by the pipeline. Some manual annotation was performed, to extract sex or age where these were not included in the data set metadata but were clear from the description of the project. For example, BioProject PRJNA451072 involves an analysis of boar taint and by definition must have used males. Testis samples are available for the same BioProject but the metadata do not state that the samples were male. It is neither practical nor desirable to manually review the metadata for all available RNAseq data sets. Other libraries could have been incorporated had adequate metadata been available to the selection pipeline. We urge submitters to make the metadata as comprehensive as possible so that the maximum value can be extracted from their studies and the excessive use of experimental animals and unnecessary duplication can be avoided. The Functional Annotation of Animal Genomes (FAANG) community (Andersson et al., 2015; https://www.faang.org/) have published guidelines for metadata and developed tools to assist with validating metadata and data for submission to public data repositories (Harrison et al., 2018). The FAANG Data Portal (https://data.faang.org/home) seeks to catalogue functional genomic data sets for pigs and other domesticated animals and capture both high quality metadata and protocols followed in the generation of the FAANG data sets.

### A Resource and a Guide to Functional Annotation

Curation of individual unannotated novel ENSSSCG transcripts in the CNS cluster (cluster 4) of the main atlas revealed that the large majority of these gene models are likely to be artefactual due to incomplete coverage of alternative 5', 3' and internal variable exons with RNA expression data. No doubt continued generation and analysis of full length RNAseq data in humans (Anvar et al., 2018) and pigs (Beiki et al., 2019) and comparative analysis across other livestock species (Giuffra et al., 2019) will lead to both functional annotation of such variation and compression of transcriptional units (Dawson et al., 2017). Some of this will be achieved by merging annotations on the two pig genomes currently available on Ensembl and revising the index used by Kallisto accordingly. Coexpression between unannotated “novel genes” and neighbouring genes in the same orientation can also provide a preliminary indication that both may be products of the same transcriptional unit. For example, one stringently microglia-associated transcript in mouse, rat and human (Carpanini et al., 2017; Pridans et al., 2018; Patir et al., 2019) that we did not detect at all in pig CNS or other data sets is *SELPLG.* There was no annotated *SELPLG* gene, or even an ambiguous ENSSSCG ID in Sscrofa11.1 whereas *SELPLG* was annotated with several alternative transcripts orthologous to the human gene on USMARCv1.0*. OLFML3*, another important marker of microglia in the mouse and human brain (Neidert et al., 2018) was not annotated on Sscrofa11.1 because it had been merged with the adjacent *HIPK1* transcript, whereas it was correctly assigned on USMARCv1.0. Since downloading the data sets used in this analysis, further releases of Ensembl (Ensembl 97/98) have been made. **Table S5** provides a list of unannotated genes that currently (November 2019) appear to have distinct descriptions and/or symbols in Ensembl and NCBI. The orthology criteria are described in the table.

One of the main applications for an expression atlas is in the transition from phenotype and genotype data to likely causation; the prioritization of candidate genes in genomic intervals identified by genome-wide association studies. To take just one example, candidate intervals associated with susceptibility to boar taint (Rowe et al., 2014) might contain genes that are coexpressed in the liver with the strong functional candidate *CYP2E1.* This prediction was explored and confirmed by Drag et al. (2017), who generated one of the datasets integrated here. Our atlas differs from the current pig expression atlas (https://www.ebi.ac.uk/gxa) in that automated selection makes use, where possible, of all available data rather than a curated subset of chosen libraries. By contrast, the EBI Expression Atlas for pig currently contains only one “baseline” data set, from FAANG (BioProject PRJEB19386). The atlas created for our study includes those data supplemented with many more samples and additional tissues which adds considerable power to detect correlated expression. We have created a data viewer on BioGPS (http://biogps.org/pigatlas) which also hosts the previous microarray-based expression atlas (Freeman et al., 2012) allowing some cross-over validation, as well as human and mouse baseline sets. For comparative analysis, BioGPS also displays our previous atlas projects for sheep (http://biogps.org/sheepatlas) and chicken (http://biogps.org/chickenatlas). The averaged processed primary expression data output from Kallisto used in the generation of the atlas is provided in **Table S6**. These data can be uploaded into Graphia to regenerate clusters using different threshholds. The data set can also be supplemented with additional data sets from new projects down-sized using the same pipeline and reclustered using Graphia.

The analysis of the clusters generated using our pipeline strongly supports the principle of guilt-by-association and the validity of the data sampling approach. The same approach was applied in the generation of a chicken atlas (Bush et al., 2018) and can be applied to any species for which there are substantial data sets in the public domain. The analysis we have presented for the pig transcriptome and the atlas that has been generated is comprehensive but it is also ephemeral. The stringency of coexpression depends upon the number and diversity of samples available and thresholds can be varied to identify more stringent associations. It will be relatively straightforward in the future to add more samples from diverse tissues, developmental stages or physiological states to provide an additional resource for the study of pig genetics and functional genomics.

## Data Availability Statement

All datasets generated for this study are included in the article/**Supplementary Material**. Details of samples extracted from the public domain are included in the **Supplementary Material**.

## Author Contributions

SB developed the informatics pipeline and generated the primary expression data. KS performed the network analyses. DH wrote the manuscript and contributed to informatic analysis. SB, KS, and AA contributed to manuscript editing. AA, CM, EC, HF, LW, LE, and RT generated and analyzed primary RNAseq data. CW and AS generated the BioGPS visualization.

## Funding

The Roslin Institute receives core strategic funding from the Biotechnology and Biological Sciences Research Council, UK (grant numbers BB/J004235/1, BBS/E/D/20211550, BBS/E/D/20211552, BBS/E/D/10002070). DH and KS are supported by the Mater Foundation and the Translational Research Institute which received funding from the Australian Government. The generation of the BioGPS resource was supported by R01GM083924 (NIH NIGMS) to CW and AS.

## Conflict of Interest

The authors declare that the research was conducted in the absence of any commercial or financial relationships that could be construed as a potential conflict of interest.

## References

[B1] Andersson L. Archibald A. L. Bottema C. D. Brauning R. Burgess S. C. Burt D. W. (2015). Coordinated international action to accelerate genome-to-phenome with FAANG, the Functional Annotation of Animal Genomes project. Genome Biol. 16, 57. 10.1186/s13059-015-0622-4 25854118 PMC4373242

[B2] Anvar S. Y. Allard G. Tseng E. Sheynkman G. M. de Klerk E. Vermaat M. (2018). ‘Full-length mRNA sequencing uncovers a widespread coupling between transcription initiation and mRNA processing’. Genome Biol. 19, 46. 10.1186/s13059-018-1418-0 29598823 PMC5877393

[B3] Ballouz S. Weber M. Pavlidis P. Gillis J. (2017). ‘EGAD: ultra-fast functional analysis of gene networks’. Bioinformatics 33, 612–614. 10.1093/bioinformatics/btw695 27993773 PMC6041978

[B4] Bedont J. L. LeGates T. A. Buhr E. Bathini A. Ling J. P. Bell B. (2017). ‘An LHX1-Regulated Transcriptional Network Controls Sleep/Wake Coupling and Thermal Resistance of the Central Circadian Clockworks’. Curr. Biol. 27, 128–136. 10.1016/j.cub.2016.11.008 28017605 PMC5269403

[B5] Beiki H. Liu H. Huang J. Manchanda N. Nonneman D. Smith T. P. L. (2019). ‘Improved annotation of the domestic pig genome through integration of Iso-Seq and RNA-seq data’. BMC Genomics 20, 344. 10.1186/s12864-019-5709-y 31064321 PMC6505119

[B6] Bray N. L. Pimentel H. Melsted P. Pachter L. (2016). ‘Near-optimal probabilistic RNA-seq quantification’. Nat. Biotechnol. 34, 525–527. 10.1038/nbt.3519 27043002

[B7] Burkard C. Opriessnig T. Mileham A. J. Stadejek T. Ait-Ali T. Lillico S. G. (2018). ‘Pigs Lacking the Scavenger Receptor Cysteine-Rich Domain 5 of CD163 Are Resistant to Porcine Reproductive and Respiratory Syndrome Virus 1 Infection’. J. Virol 92, e00415–8. 10.1128/JVI.00415-18 29925651 PMC6069206

[B8] Bush S. J. Freem L. MacCallum A. J. O'Dell J. Wu C. Afrasiabi C. (2018). ‘Combination of novel and public RNA-seq datasets to generate an mRNA expression atlas for the domestic chicken’. BMC Genomics 19, 594. 10.1186/s12864-018-4972-7 30086717 PMC6081845

[B9] Butler J. E. Lager K. M. Splichal I. Francis D. Kacskovics I. Sinkora M. (2009). ‘The piglet as a model for B cell and immune system development’. Vet Immunol. Immunopathol 128, 147–170. 10.1016/j.vetimm.2008.10.321 19056129 PMC2828348

[B10] Carpanini S. M. Wishart T. M. Gillingwater T. H. Manson J. C. Summers K. M. (2017). ‘Analysis of gene expression in the nervous system identifies key genes and novel candidates for health and disease’. Neurogenetics 18, 81–95. 10.1007/s10048-017-0509-5 28190221 PMC5359387

[B11] Castinetti F. Brinkmeier M. L. Mortensen A. H. Vella K. R. Gergics P. Brue T. (2015). ‘ISL1 Is Necessary for Maximal Thyrotrope Response to Hypothyroidism’. Mol. Endocrinol. 29, 1510–1521. 10.1210/me.2015-1192 26296153 PMC4588728

[B12] Charles M. A. Saunders T. L. Wood W. M. Owens K. Parlow A. F. Camper S. A. (2006). ‘Pituitary-specific Gata2 knockout: effects on gonadotrope and thyrotrope function’. Mol. Endocrinol. 20, 1366–1377. 10.1210/me.2005-0378 16543408

[B13] Chitu V. Stanley E. R. (2017). ‘Regulation of Embryonic and Postnatal Development by the CSF-1 Receptor’. Curr. Top. Dev. Biol. 123, 229–275. 10.1016/bs.ctdb.2016.10.004 28236968 PMC5479137

[B14] Clark E. L. Bush S. J. McCulloch M. E. B. Farquhar I. L. Young R. Lefevre L. (2017). ‘A high resolution atlas of gene expression in the domestic sheep (Ovis aries)’. PloS Genet. 13, e1006997. 10.1371/journal.pgen.1006997 28915238 PMC5626511

[B15] Conforto T. L. Zhang Y. Sherman J. Waxman D. J. (2012). ‘Impact of CUX2 on the female mouse liver transcriptome: activation of female-biased genes and repression of male-biased genes’. Mol. Cell Biol. 32, 4611–4627. 10.1128/MCB.00886-12 22966202 PMC3486175

[B16] Curwen V. Eyras E. Andrews T. D. Clarke L. Mongin E. Searle S. M. (2004). ‘The Ensembl automatic gene annotation system’. Genome Res. 14, 942–950. 10.1101/gr.1858004 15123590 PMC479124

[B17] Dawson H. D. Loveland J. E. Pascal G. Gilbert J. G. Uenishi H. Mann K. M. (2013). ‘Structural and functional annotation of the porcine immunome’. BMC Genomics 14, 332. 10.1186/1471-2164-14-332 23676093 PMC3658956

[B18] Dawson H. D. Chen C. Gaynor B. Shao J. Urban J. F. (2017). ‘The Porcine Translational Research Database: a Manually Curated, Genomics and Proteomics-Based Research Resource’. BMC Genomics 18, 643. 10.1186/s12864-017-4009-7 28830355 PMC5568366

[B19] Determan C. Jr. Anderson R. Becker A. Witowski N. Lusczek E. Mulier K. (2014). ‘Fed state prior to hemorrhagic shock and polytrauma in a porcine model results in altered liver transcriptomic response’. PloS One 9, e100088. 10.1371/journal.pone.0100088 24937255 PMC4061062

[B20] Drag M. Skinkyte-Juskiene R. Do D. N. Kogelman L. J. Kadarmideen H. N. (2017). Differential expression and co-expression networks reveal candidate biomarkers of boar taint in non-castrated pigs. Sci. Rep. 7, 12205. 10.1038/s41598-017-11928-0 28939879 PMC5610188

[B21] Drouin J. (2016). ‘60 YEARS OF POMC: Transcriptional and epigenetic regulation of POMC gene expression’. J. Mol. Endocrinol. 56, T99–T112. 10.1530/JME-15-0289 26792828

[B22] Fairbairn L. Kapetanovic R. Sester D. P. Hume D. A. (2011). ‘The mononuclear phagocyte system of the pig as a model for understanding human innate immunity and disease’. J. Leukoc Biol. 89, 855–871. 10.1189/jlb.1110607 21233410

[B23] Fairbairn L. Kapetanovic R. Beraldi D. Sester D. P. Tuggle C. K. Archibald A. L. (2013). ‘Comparative analysis of monocyte subsets in the pig’. J. Immunol. 190, 6389–6396. 10.4049/jimmunol.1300365 23667115

[B24] FANTOM Consortium Forrest A. R. Kawaji H. Rehli M. Baillie J. K. de Hoon M. J. (2014). ‘A promoter-level mammalian expression atlas’. Nature 507, 462–470. 10.1038/nature13182 24670764 PMC4529748

[B25] Freeman T. C. Ivens A. Baillie J. K. Beraldi D. Barnett M. W. Dorward D. (2012). ‘A gene expression atlas of the domestic pig’. BMC Biol. 10, 90. 10.1186/1741-7007-10-90 23153189 PMC3814290

[B26] Gillis J. Pavlidis P. (2012). “Guilt by association” is the exception rather than the rule in gene networks’. PloS Comput. Biol. 8, e1002444. 10.1371/journal.pcbi.1002444 22479173 PMC3315453

[B27] Giotti B. Chen S. H. Barnett M. W. Regan T. Ly T. Wiemann S. (2018). ‘Assembly of a Parts List of the Human Mitotic Cell Cycle Machinery’. J. Mol. Cell Biol 11, 703–718. 10.1093/jmcb/mjy063 PMC6788831 30452682

[B28] Giuffra E. Tuggle C. K. FAANG Consortium (2019). ‘Functional Annotation of Animal Genomes (FAANG): Current Achievements and Roadmap’. Annu. Rev. Anim Biosci 7, 65–88. 10.1146/annurev-animal-020518-114913 30427726

[B29] GTEx Consortium (2015). ‘Human genomics. The Genotype-Tissue Expression (GTEx) pilot analysis: multitissue gene regulation in humans’. Science 348, 648–660. 10.1126/science.1262110 25954001 PMC4547484

[B30] Gutierrez K. Dicks N. Glanzner W. G. Agellon L. B. Bordignon V. (2015). ‘Efficacy of the porcine species in biomedical research’. Front. Genet. 6, 293. 10.3389/fgene.2015.00293 26442109 PMC4584988

[B31] Harrison P. W. Fan J. Richardson D. Clarke L. Zerbino D. Cochrane G. (2018). FAANG, estbalishing metadata standards, validation and best practices for the farmed and companion animal community. Anim. Genet. 49, 520–526. 10.1111/age.12736 30311252 PMC6334167

[B32] Hasenfuss G. (1998). ‘Animal models of human cardiovascular disease, heart failure and hypertrophy’. Cardiovasc. Res. 39, 60–76. 10.1016/s0008-6363(98)00110-2 9764190

[B33] Hume D. A. Summers K. M. Raza S. Baillie J. K. Freeman T. C. (2010). ‘Functional clustering and lineage markers: insights into cellular differentiation and gene function from large-scale microarray studies of purified primary cell populations’. Genomics 95, 328–338. 10.1016/j.ygeno.2010.03.002 20211243

[B34] Jubb A. W. Young R. S. Hume D. A. Bickmore W. A. (2016). ‘Enhancer Turnover Is Associated with a Divergent Transcriptional Response to Glucocorticoid in Mouse and Human Macrophages’. J. Immunol. 196, 813–822. 10.4049/jimmunol.1502009 26663721 PMC4707550

[B35] Kapetanovic R. Fairbairn L. Beraldi D. Sester D. P. Archibald A. L. Tuggle C. K. (2012). ‘Pig bone marrow-derived macrophages resemble human macrophages in their response to bacterial lipopolysaccharide’. J. Immunol. 188, 3382–3394. 10.4049/jimmunol.1102649 22393154

[B36] Kapetanovic R. Fairbairn L. Downing A. Beraldi D. Sester D. P. Freeman T. C. (2013). ‘The impact of breed and tissue compartment on the response of pig macrophages to lipopolysaccharide’. BMC Genomics 14, 581. 10.1186/1471-2164-14-581 23984833 PMC3766131

[B37] Lau-Corona D. Suvorov A. Waxman D. J. (2017). ‘Feminization of Male Mouse Liver by Persistent Growth Hormone Stimulation: Activation of Sex-Biased Transcriptional Networks and Dynamic Changes in Chromatin States’. Mol. Cell Biol. 37, e00301–e00317. 10.1128/MCB.00301-17 28694329 PMC5599723

[B38] Li Y. Fang C. Fu Y. Hu A. Li C. Zou C. (2018). A survey of transcriptome complexity in Sus Scofa using single molecule long-read sequencing. DNA Res. 25, 421–437. 10.1093/dnares/dsy014 29850846 PMC6105124

[B39] Mabbott N. A. Baillie J. K. Brown H. Freeman T. C. Hume D. A. (2013). ‘An expression atlas of human primary cells: inference of gene function from coexpression networks’. BMC Genomics 14, 632. 10.1186/1471-2164-14-632 24053356 PMC3849585

[B40] Mamillapalli R. Wysolmerski J. (2010). ‘The calcium-sensing receptor couples to Galpha(s) and regulates PTHrP and ACTH secretion in pituitary cells’. J. Endocrinol. 204, 287–297. 10.1677/JOE-09-0183 20032198 PMC3777408

[B41] Neidert N. von Ehr A. Zoller T. Spittau B. (2018). ‘Microglia-Specific Expression of Olfml3 Is Directly Regulated by Transforming Growth Factor beta1-Induced Smad2 Signaling’. Front. Immunol. 9, 1728. 10.3389/fimmu.2018.01728 30093905 PMC6070609

[B42] Papatheodorou I. Fonseca N. A. Keays M. Tang Y. A. Barrera E. Bazant W. (2018). ‘Expression Atlas: gene and protein expression across multiple studies and organisms’. Nucleic Acids Res. 46, D246–DD51. 10.1093/nar/gkx1158 29165655 PMC5753389

[B43] Patir A. Shih B. McColl B. W. Freeman T. C. (2019). ‘A core transcriptional signature of human microglia: Derivation and utility in describing region-dependent alterations associated with Alzheimer’s disease’. Glia 67, 1240–1253. 10.1002/glia.23572 30758077

[B44] Perleberg C. Kind A. Schnieke A. (2018). ‘Genetically engineered pigs as models for human disease’. Dis. Model Mech. 11, dmm030783. 10.1242/dmm.030783 29419487 PMC5818075

[B45] Pridans C. Raper A. Davis G. M. Alves J. Sauter K. A. Lefevre L. (2018). ‘Pleiotropic Impacts of Macrophage and Microglial Deficiency on Development in Rats with Targeted Mutation of the Csf1r Locus’. J. Immunol. 201, 2683–2699. 10.4049/jimmunol.1701783 30249809 PMC6196293

[B46] Robert C. Kapetanovic R. Beraldi D. Watson M. Archibald A. L. Hume D. A. (2015). ‘Identification and annotation of conserved promoters and macrophage-expressed genes in the pig genome’. BMC Genomics 16, 970. 10.1186/s12864-015-2111-2 26582032 PMC4652390

[B47] Romanov R. A. Alpar A. Zhang M. D. Zeisel A. Calas A. Landry M. (2015). ‘A secretagogin locus of the mammalian hypothalamus controls stress hormone release’. EMBO J. 34, 36–54. 10.15252/embj.201488977 25430741 PMC4291479

[B48] Rowe S. J. Karacaoren B. de Koning D. J. Lukic B. Hastings-Clark N. Velander I. (2014). “Analysis of the genetics of boar taint reveals both single SNPs and regional effects”. BMC Genomics 14, 424. 10.1186/1471-2164-15-424 PMC4059876 24894739

[B49] Sauter K. A. Waddell L. A. Lisowski Z. M. Young R. Lefevre L. Davis G. M. (2016). ‘Macrophage colony-stimulating factor (CSF1) controls monocyte production and maturation and the steady-state size of the liver in pigs’. Am. J. Physiol. Gastrointest Liver Physiol. 311, G533–G547. 10.1152/ajpgi.00116.2016 27445344 PMC5076001

[B50] Schroder K. Irvine K. M. Taylor M. S. Bokil N. J. Le Cao K. A. Masterman K. A. (2012). ‘Conservation and divergence in Toll-like receptor 4-regulated gene expression in primary human versus mouse macrophages’. Proc. Natl. Acad. Sci. U.S.A. 109, E944–E953. 10.1073/pnas.1110156109 22451944 PMC3341041

[B51] Scott C. L. T’Jonck W. Martens L. Todorov H. Sichien D. Soen B. (2018). ‘The Transcription Factor ZEB2 Is Required to Maintain the Tissue-Specific Identities of Macrophages’. Immunity 49, 312–25 e5. 10.1016/j.immuni.2018.07.004 30076102 PMC6104815

[B52] Singh A. J. Ramsey S. A. Filtz T. M. Kioussi C. (2018). ‘Differential gene regulatory networks in development and disease’. Cell Mol. Life Sci. 75, 1013–1025. 10.1007/s00018-017-2679-6 29018868 PMC11105524

[B53] Sobrier M. L. Tsai Y. C. Perez C. Leheup B. Bouceba T. Duquesnoy P. (2016). ‘Functional characterization of a human POU1F1 mutation associated with isolated growth hormone deficiency: a novel etiology for IGHD’. Hum. Mol. Genet. 25, 472–483. 10.1093/hmg/ddv486 26612202 PMC5007599

[B54] Summers K. M. Raza S. van Nimwegen E. Freeman T. C. Hume D. A. (2010). ‘Co-expression of FBN1 with mesenchyme-specific genes in mouse cell lines: implications for phenotypic variability in Marfan syndrome’. Eur. J. Hum. Genet. 18, 1209–1215. 10.1038/ejhg.2010.91 20551991 PMC2987476

[B55] Theocharidis A. van Dongen S. Enright A. J. Freeman T. C. (2009). ‘Network visualization and analysis of gene expression data using BioLayout Express(3D)’. Nat. Protoc. 4, 1535–1550. 10.1038/nprot.2009.177 19798086

[B56] Trapnell B. C. Nakata K. Bonella F. Campo I. Griese M. Hamilton J. (2019). ‘Pulmonary alveolar proteinosis’. Nat. Rev. Dis. Primers 5, 16. 10.1038/s41572-019-0066-3 30846703

[B57] van Dongen S. Abreu-Goodger C. (2012). “‘Using MCL to extract clusters from networks.’,” in Bacterial molecular networks: Methods and protocols. Eds. Van Helden J. Toussaint A. Theiffry D. (New York, NY, USA: Springer). 10.1007/978-1-61779-361-5_15 22144159

[B58] Villar D. Berthelot C. Aldridge S. Rayner T. F. Lukk M. Pignatelli M. (2015). ‘Enhancer evolution across 20 mammalian species’. Cell 160, 554–566. 10.1016/j.cell.2015.01.006 25635462 PMC4313353

[B59] Waddell L. A. Lefevre L. Bush S. J. Raper A. Young R. Lisowski Z. M. (2018). ‘ADGRE1 (EMR1, F4/80) Is a Rapidly-Evolving Gene Expressed in Mammalian Monocyte-Macrophages’. Front. Immunol. 9, 2246. 10.3389/fimmu.2018.02246 30327653 PMC6174849

[B60] Wang J. Gamazon E. R. Pierce B. L. Stranger B. E. Im H. K. Gibbons R. D. (2016). ‘Imputing Gene Expression in Uncollected Tissues Within and Beyond GTEx’. Am. J. Hum. Genet. 98, 697–708. 10.1016/j.ajhg.2016.02.020 27040689 PMC4833292

[B61] Wang S. Stanika R. I. Wang X. Hagen J. Kennedy M. B. Obermair G. J. (2017). ‘Densin-180 Controls the Trafficking and Signaling of L-Type Voltage-Gated Cav1.2 Ca(2+) Channels at Excitatory Synapses’. J. Neurosci. 37, 4679–4691. 10.1523/JNEUROSCI.2583-16.2017 28363979 PMC5426563

[B62] Warr A. Affara N. Aken B. Bickhart D. M. Billis K. Chow W. (2019). ‘An improved pig reference genome sequence to enable pig genetics and genomics research’. BioRxiv, 563007, 668921. 10.1101/668921 PMC7448572 32543654

[B63] Whitelaw C. B. Sheets T. P. Lillico S. G. Telugu B. P. (2016). ‘Engineering large animal models of human disease’. J. Pathol. 238, 247–256. 10.1002/path.4648 26414877 PMC4737318

[B64] Xie H. Hoffmann H. M. Meadows J. D. Mayo S. L. Trang C. Leming S. S. (2015). ‘Homeodomain Proteins SIX3 and SIX6 Regulate Gonadotrope-specific Genes During Pituitary Development’. Mol. Endocrinol. 29, 842–855. 10.1210/me.2014-1279 25915183 PMC4447639

[B65] Young R. Bush S. J. Lefevre L. McCulloch M. E. B. Lisowski Z. M. Muriuki C. (2018). ‘Species-Specific Transcriptional Regulation of Genes Involved in Nitric Oxide Production and Arginine Metabolism in Macrophages’. Immunohorizons 2, 27–37. 10.4049/immunohorizons.1700073 30467554 PMC6245571

[B66] Young R. Lefevre L. Bush S. J. Joshi. A. Singh S. H. jadhav S. K. (2019). A gene expression atlas of the domestic water buffalo (*Bubalus bubalis*). Front. Genet. 10, 688. 10.3389/fgene.2019.00668 31428126 PMC6689995

[B67] Zhang Y. Klein K. Sugathan A. Nassery N. Dombkowski A. Zanger U. M. (2011). ‘Transcriptional profiling of human liver identifies sex-biased genes associated with polygenic dyslipidemia and coronary artery disease’. PloS One 6, e23506. 10.1371/journal.pone.0023506 21858147 PMC3155567

[B68] Zhu Y. Stephens R. M. Meltzer P. S. Davis S. R. (2013). ‘SRAdb: query and use public next-generation sequencing data from within R’. BMC Bioinf. 14, 19. 10.1186/1471-2105-14-19 PMC3560148 23323543

