# Supplementary material for: Functional Annotation of the Transcriptome of the Pig, Sus scrofa, Based Upon Network Analysis of an RNAseq Transcriptional Atlas
Source: Front Genet. 2020 Feb 14;10:1355. doi: 10.3389/fgene.2019.01355 (PMC7034361; doi:10.3389/fgene.2019.01355)
Supplement: Supplementary file 1 [file Presentation_1.pdf]

Functional annotation of the transcriptome of the pig, *Sus scrofa*, based upon network analysis of an RNAseq transcriptional atlas.

Kim M Summers, Stephen J Bush, Chunlei Wu, Andrew I. Su, Charity Muriuki, Emily L Clark, Heather A. Finlayson, Lel Eory, Lindsey A Waddell, Richard Talbot, Alan L Archibald, David A Hume

Supplementary Figures

Figure S1. **Correlation graph settings for network analysis with Graphia Professional**
Figure S1A. Settings for the overall atlas.
Figure S1B. Settings for the analysis of liver samples.
Figure S1C. Settings for the analysis of central nervous system samples.
Figure S2. **Sample to sample networks**
Figure S2A. Networks generated for the overall atlas.
Figure S2B. Analysis of liver samples.
Figure S2C. Analysis of central nervous system samples.
References for Supplementary Figures

Next Page

Figure S1. Correlation graph settings for network analysis with Graphia Professional
The correlation threshold for each gene-to-gene analysis was chosen to optimise the number of nodes and edges in order to generate meaningful clusters.
A. Settings for the overall atlas.
B. Settings for the analysis of liver samples.
C. Settings for the analysis of central nervous system samples.

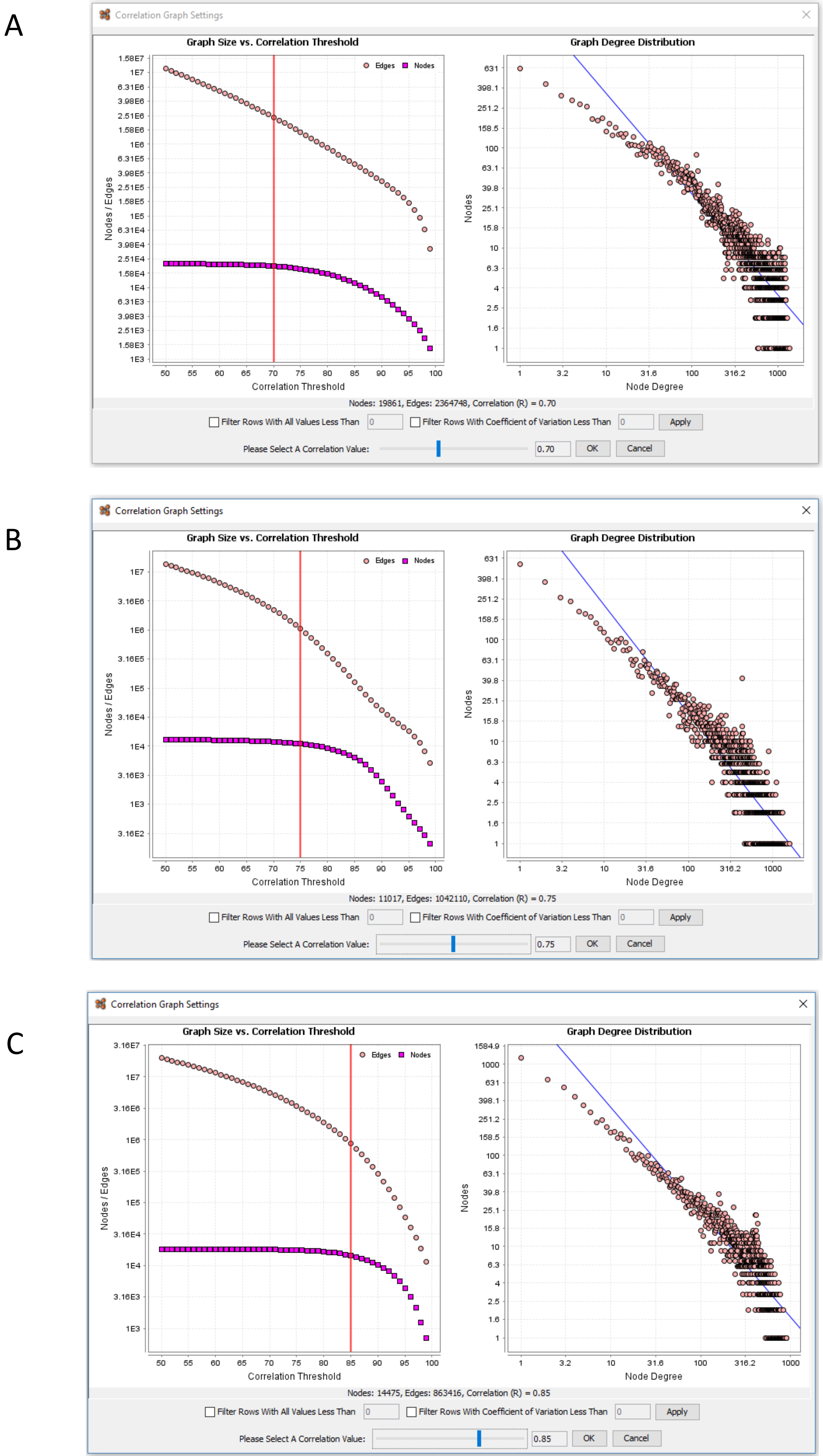
A
Correlation Graph Settings
Graph Size vs. Correlation Threshold
Edges
Nodes
Nodes / Edges
Correlation Threshold
Graph Degree Distribution
Nodes
Node Degree
Nodes: 19861, Edges: 2364748, Correlation (R) = 0.70
Filter Rows With All Values Less Than
Filter Rows With Coefficient of Variation Less Than
Apply
Please Select A Correlation Value:
0.70
OK
Cancel
B
Correlation Graph Settings
Graph Size vs. Correlation Threshold
Edges
Nodes
Nodes / Edges
Correlation Threshold
Graph Degree Distribution
Nodes
Node Degree
Nodes: 11017, Edges: 1042110, Correlation (R) = 0.75
Filter Rows With All Values Less Than
Filter Rows With Coefficient of Variation Less Than
Apply
Please Select A Correlation Value:
0.75
OK
Cancel
C
Correlation Graph Settings
Graph Size vs. Correlation Threshold
Edges
Nodes
Nodes / Edges
Correlation Threshold
Graph Degree Distribution
Nodes
Node Degree
Nodes: 14475, Edges: 863416, Correlation (R) = 0.85
Filter Rows With All Values Less Than
Filter Rows With Coefficient of Variation Less Than
Apply
Please Select A Correlation Value:
0.85
OK
Cancel

Figure S2. Sample to sample networks

A. Networks generated for the overall atlas. Colours show the organ system of the samples. Upper panel shows the result for all nodes (samples) at R ≥ 0.38; lower panel shows the network at R ≥ 0.85, which clusters samples of related origin together. Note that muscle, heart, spleen, liver and testis samples from BioProject PRJNA297855 are separated from the other samples of the same tissues. These samples are from a study of Guizhou minipigs which may be very different from the full sized breeds of the other samples.
B. Network generated for the liver analysis. Colours indicate the BioProject from which the samples were drawn.
C. Network generated for the central nervous system analysis.

A. Overall atlas

Node colour	Organ system
	Cardiovascular system
	Endocrine system
	Female reproductive system
	Gastrointestinal tract
	Immune system
	Integumentary system
	Male reproductive system
	Mixture of multiple tissues
	Muscular system
	Nervous system
	Cells, cell lines, embryo
	Renal system
	Respiratory system

Sample-to-sample analysis by organ system
r ≥ 0.38
206 nodes, 14,243 edges

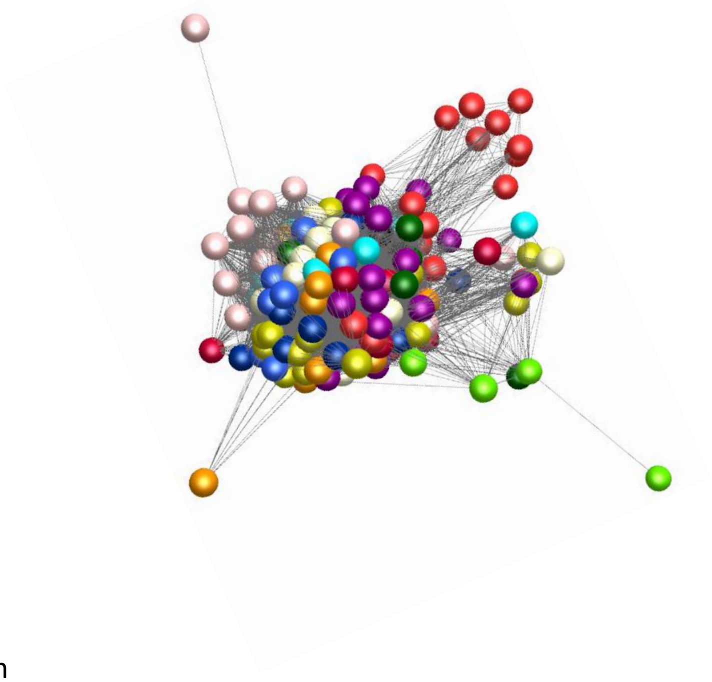

Sample-to-sample analysis by organ system
r ≥ 0.85
188 nodes, 1378 edges

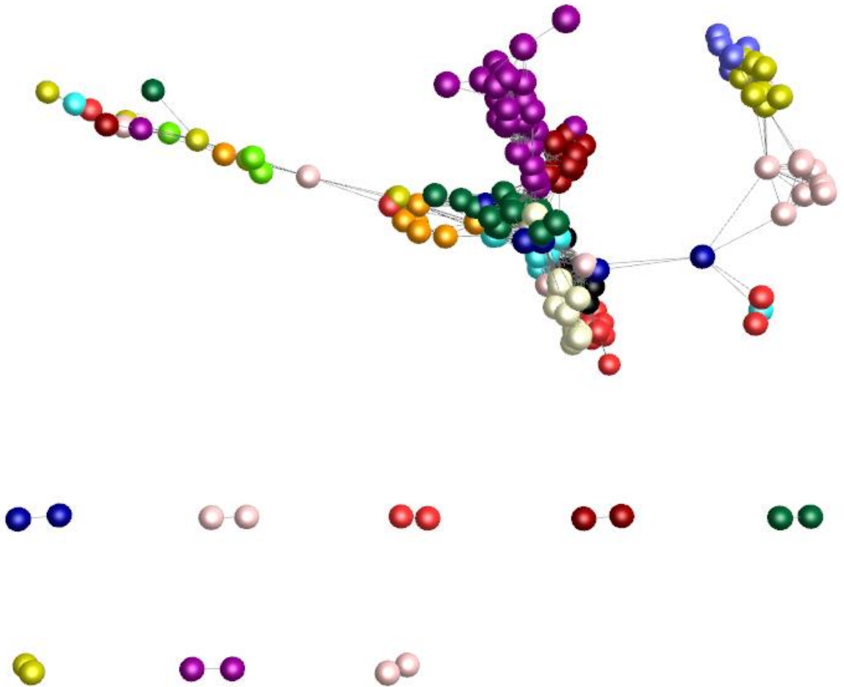

B. Analysis of liver samples

r ≥ 0.9
102 nodes, 1301 edges
Nodes are coloured by BioProject ID

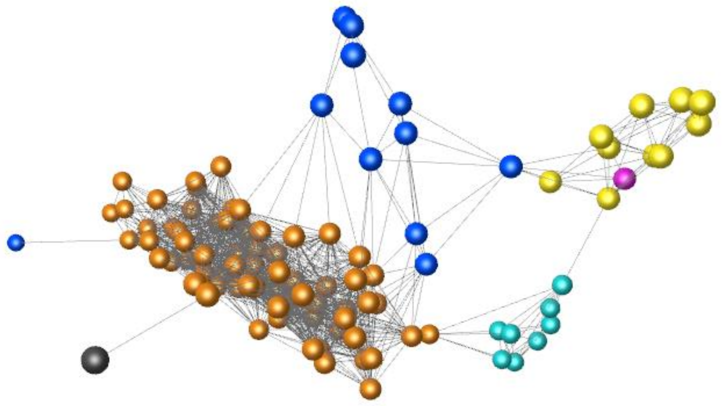

Node colour	BioProject	Description
	PRJNA193204	Berkshire pig, adult (Sodhi et al., 2017)
	PRJNA240332	Yorkshire-Landrace cross pigs, juvenile (15-20 kg), haemorrhagic shock model (Determan et al., 2014)
	PRJNA297855	Guizhou minipig, 240 days postnatal (Tang et al., 2017)
	PRJNA305529	Iberian-Landrace cross, backcrossed to Landrace, 175 days postnatal, low and high growth and fatness (Martinez-Montes 2016)
	PRJNA311523	Various breeds, 60 days postnatal {Li 2017}
	PRJNA451072	Danish Landrace pigs, adult (100 kg), high and medium boar taint (Drag et al., 2017)

C. Analysis of central nervous system samples

r ≥ 0.87
30 nodes, 142 edges
Nodes are coloured by region

Node colour	Region
	Amygdala
	Brain
	Brainstem
	Corpus callosum
	Cortex
	Thalamus
	Forebrain
	Frontal cortex
	Hippocampal formation
	Hypothalamus
	Medulla oblongata
	Occipital cortex
	Pituitary gland
	Pons

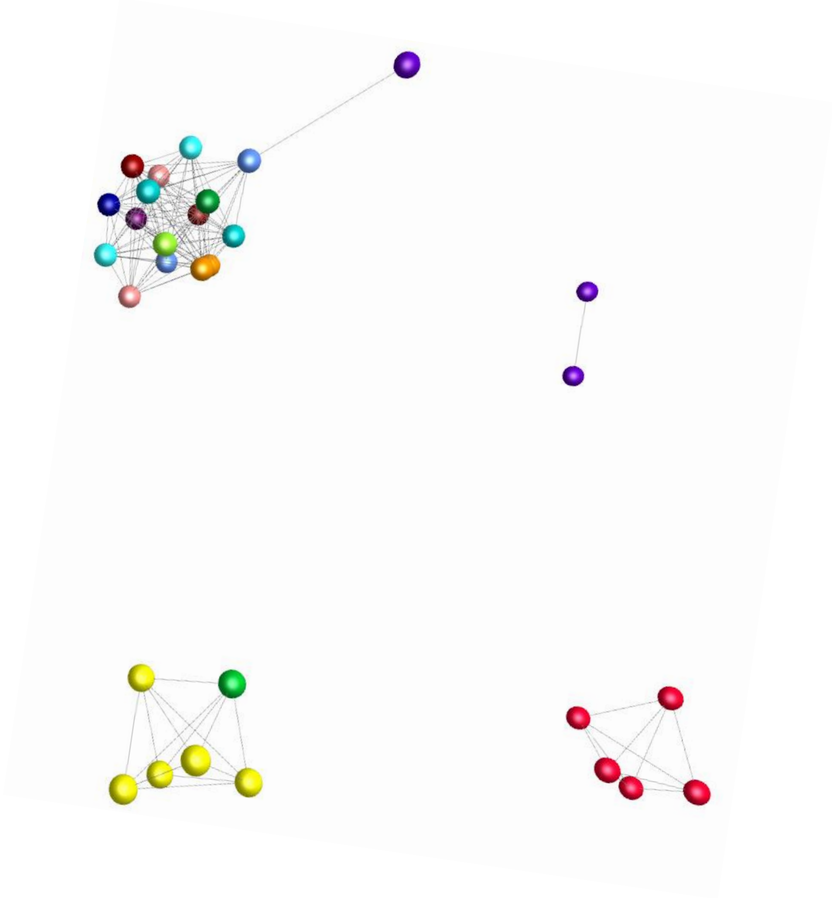

References for Supplementary Figures

Determan, C., Jr., R. Anderson, A. Becker, N. Witowski, E. Lusczek, K. Mulier et al. (2014). Fed state prior to hemorrhagic shock and polytrauma in a porcine model results in altered liver transcriptomic response. PLoS One **9**(6): e100088.

Drag, M., R. Skinkyte-Juskiene, D. N. Do, L. J. A. Kogelman and H. N. Kadarmideen (2017). Differential expression and co-expression gene networks reveal candidate biomarkers of boar taint in non-castrated pigs. Sci Rep **7**(1): 12205.

Li, M., L. Chen, S. Tian, Y. Lin, Q. Tang, X. Zhou, et al. (2017). Comprehensive variation discovery and recovery of missing sequence in the pig genome using multiple de novo assemblies. Genome Res **27**(5): 865-874.

Martinez-Montes, A. M., A. Fernandez, D. Perez-Montarelo, E. Alves, R. M. Benitez, Y. Nunez, et al. (2017). Using RNA-Seq SNP data to reveal potential causal mutations related to pig production traits and RNA editing. Anim Genet **48**(2): 151-165.

Sodhi, S. S., K. D. Song, M. Ghosh, N. Sharma, S. J. Lee, J. H. Kim, et al. (2014). Comparative transcriptomic analysis by RNA-seq to discern differential expression of genes in liver and muscle tissues of adult Berkshire and Jeju Native Pig. Gene **546**(2): 233-242.

Tang, Z., Y. Wu, Y. Yang, Y. T. Yang, Z. Wang, J. Yuan, et al. (2017). "Comprehensive analysis of long non-coding RNAs highlights their spatio-temporal expression patterns and evolutional conservation in Sus scrofa." Sci Rep **7**: 43166.
